# Within-host competition can delay evolution of drug resistance in malaria

**DOI:** 10.1371/journal.pbio.2005712

**Published:** 2018-08-21

**Authors:** Mary Bushman, Rustom Antia, Venkatachalam Udhayakumar, Jacobus C. de Roode

**Affiliations:** 1 Department of Biology, Emory University, Atlanta, Georgia, United States of America; 2 Malaria Branch, Division of Parasitic Diseases and Malaria, Center for Global Health, Centers for Disease Control and Prevention, Atlanta, Georgia, United States of America; Imperial College London, United Kingdom of Great Britain and Northern Ireland

## Abstract

In the malaria parasite *P*. *falciparum*, drug resistance generally evolves first in low-transmission settings, such as Southeast Asia and South America. Resistance takes noticeably longer to appear in the high-transmission settings of sub-Saharan Africa, although it may spread rapidly thereafter. Here, we test the hypothesis that competitive suppression of drug-resistant parasites by drug-sensitive parasites may inhibit evolution of resistance in high-transmission settings, where mixed-strain infections are common. We employ a cross-scale model, which simulates within-host (infection) dynamics and between-host (transmission) dynamics of sensitive and resistant parasites for a population of humans and mosquitoes. Using this model, we examine the effects of transmission intensity, selection pressure, fitness costs of resistance, and cross-reactivity between strains on the establishment and spread of resistant parasites. We find that resistant parasites, introduced into the population at a low frequency, are more likely to go extinct in high-transmission settings, where drug-sensitive competitors and high levels of acquired immunity reduce the absolute fitness of the resistant parasites. Under strong selection from antimalarial drug use, however, resistance spreads faster in high-transmission settings than low-transmission ones. These contrasting results highlight the distinction between establishment and spread of resistance and suggest that the former but not the latter may be inhibited in high-transmission settings. Our results suggest that within-host competition is a key factor shaping the evolution of drug resistance in *P*. *falciparum*.

## Author summary

The malaria parasite *Plasmodium falciparum* has evolved resistance to most antimalarial drugs, greatly complicating treatment and control of the disease. Curiously, although sub-Saharan Africa accounts for the majority of the global burden of malaria, the evolution of drug resistance in Africa has been markedly delayed compared to Asia and the Americas. One reason might be that, in a population in which the prevalence of infection is high, a newly emerged drug-resistant strain faces a high risk of extinction due to competition from drug-sensitive parasites that already “occupy” most of the host population. Using a mathematical model, we confirm that drug-resistant parasites face a much greater risk of extinction in a “high-transmission” setting like sub-Saharan Africa than in a “low-transmission” setting like Southeast Asia. However, we also find that when drug-resistant parasites manage to avoid extinction, their subsequent spread may be more rapid in high-transmission settings than in low-transmission settings, especially when selection is strong. These results offer a novel explanation for global patterns of drug resistance evolution in malaria and suggest a new dimension to consider in resistance prevention and containment efforts: namely, the intrinsic favorability of low- and high-transmission settings for establishment and spread of drug resistance.

## Introduction

Drug resistance is a recurring threat to effective treatment and control of *P*. *falciparum* malaria. Chloroquine—a highly effective synthetic antimalarial—was used to combat malaria on a massive scale in the 1950s; by the 1960s, resistance to chloroquine had developed in Southeast Asia and South America, and over the next few decades, chloroquine resistance spread to virtually every corner of the malaria-endemic world [1]. Sulfadoxine-pyrimethamine, the prevailing substitute for chloroquine, was also rapidly undermined by resistance [1]. Artemisinin-based combination therapies (ACTs), which are now the standard treatment for *P*. *falciparum* malaria in most countries, are in danger of widespread failure, with resistance to artemisinin and piperaquine entrenched and spreading in Southeast Asia despite focused containment efforts [2]. At present, ACTs are the last broadly effective antimalarial drugs left; the prospect of widespread resistance therefore represents a looming crisis in global health.

A substantial body of evidence indicates a strong tendency for resistance to evolve in low-transmission settings. Studies that reconstructed the evolutionary histories of resistance to chloroquine, sulfadoxine, and pyrimethamine [3–6] found that drug-resistant parasites from all over the world are frequently derived from just a handful of lineages. Furthermore, these lineages disproportionately originated in low-transmission settings, especially Southeast Asia and South America. These findings present a great puzzle: although the high-transmission settings of sub-Saharan Africa account for roughly 90% of the global burden of malaria, resistance has seldom evolved locally within Africa [7]. In at least two cases—those of resistance to chloroquine and pyrimethamine—drug-resistant genotypes were carried to Africa from Southeast Asia, after which the imported drug-resistant alleles swept across the African continent via gene flow [4,5]. Resistance mutations must occur frequently in the high-transmission settings of Africa but fail to be selected and spread through the population. This leads to the counterintuitive conclusion that low-transmission settings are more conducive to the sustained transmission of drug-resistant parasites (at least when resistance is rare)—a prerequisite for resistance to become established in the population.

There are at least three different (and nonmutually exclusive) mechanisms that could explain this counterintuitive pattern. The first is recombination during sexual reproduction in the mosquito vector, which occurs more frequently in high-transmission settings [8]. Recombination will tend to separate multiple mutations that contribute to a drug-resistant phenotype, slowing down the spread of resistance; the more loci involved, the stronger the effect will be [9]. Thus, recombination could explain the delayed appearance of multilocus resistance (such as for sulfadoxine-pyrimethamine and ACTs) in high-transmission settings [10,11]. However, recombination is unlikely to slow the spread of resistance encoded at a single locus, such as resistance to chloroquine [4,12]. The fact that both single- and multilocus resistance mechanisms show similar tendencies to evolve in low-transmission settings therefore suggests that recombination is not the primary mechanism delaying the evolution of resistance in high-transmission settings.

The second proposed mechanism is that higher levels of acquired immunity reduce selection for resistance in high-transmission settings. Clinical immunity to malaria, which reduces the likelihood of symptomatic infection, is thought to be acquired through years of frequent exposure, mainly in high-transmission settings [13]. As a result, it is believed that malaria infections are frequently asymptomatic in high-transmission settings (especially in older age groups) but generally symptomatic in low-transmission settings and that levels of antimalarial drug use will accordingly be higher in low-transmission settings, resulting in stronger selection for resistance [14, 15]. However, a 2013 meta-analysis [16] found that, although there is a positive correlation between transmission intensity and asymptomatic infections, a majority of infections are asymptomatic even in low-transmission settings, which suggests that the relationship between transmission intensity and selection for resistance is, at a minimum, weaker than previously believed. Furthermore, historical records indicate that chloroquine resistance spread rapidly across the African continent following its appearance in Kenya and Tanzania in 1978 [3], suggesting that the selection pressure was in fact adequate to drive rapid spread of resistance in Africa.

Here, we address the alternative hypothesis that within-host competition between drug-sensitive and drug-resistant parasites could inhibit the spread of resistance in high-transmission settings. Human malaria infections frequently consist of multiple parasite strains or genotypes, with higher multiplicity of infection observed in high-transmission settings [17]. Strains inhabiting the same host are limited by shared resources and strain-transcending immune responses, leading to ecological competition. Within-host competition between drug-sensitive and drug-resistant parasites has been unequivocally demonstrated in the rodent malaria parasite *P*. *chabaudi;* in this model system, drug-sensitive parasites strongly suppress the growth of drug-resistant parasites and reduce their transmission to the mosquito vector [18–22]. Empirical evidence strongly supports within-host competition in *P*. *falciparum* in humans as well [23,24]. In particular, a series of cross-sectional studies across the African continent showed that densities of chloroquine-resistant parasites were significantly reduced in children that were coinfected with chloroquine-sensitive parasites, suggesting competitive suppression [23].

To date, few mathematical models have been developed to explore the impact of within-host competition on the spread of drug resistance in malaria. Some of these have suggested that within-host competition could inhibit the spread of resistance [25,26]; however, their conclusions were likely driven by assumptions that drug resistance carried a fitness cost that was either contingent on or exacerbated by within-host competition. This essentially ensured that resistant parasites were intrinsically more affected by competition than their drug-sensitive counterparts, a finding that is not strongly supported by empirical data [23]. Thus, whether within-host competition can explain the delayed evolution of resistance in high-transmission settings on the basis of ecological competition alone, without invoking fitness costs of resistance, remains an unanswered question.

Other models have suggested that within-host competition might actually accelerate the spread of resistance in high-transmission settings because of a phenomenon known as “competitive release.” Experiments in *P*. *chabaudi* have shown that competition between sensitive and resistant parasites can be alleviated by antimalarial drug treatment, which removes the sensitive parasites from the host, allowing the resistant population to expand [18]. In *P*. *chabaudi*, competitive release increases not only the density of resistant parasites but transmission to mosquitoes as well [22,27]. Relatively simple models incorporating competitive release have shown that it can increase the rate at which resistance spreads through a population [9,28], suggesting that drug resistance should evolve more rapidly in settings with more mixed-strain infections—i.e., high-transmission settings. On the whole, empirical data contradict these predictions, but it is not immediately clear why. Competitive release has never been demonstrated in *P*. *falciparum*, so it is not known with certainty that the assumptions made in these models are valid. Alternatively, it may be that rates of antimalarial drug use are not high enough in most circumstances for the effects of competitive release to matter, especially if other factors tend to inhibit the spread of resistance in high-transmission settings. Curiously, although mathematical models have examined the effects of within-host competition and competitive release individually, no effort has been made to examine their combined effects—a significant gap, since the two almost certainly go hand in hand.

**Fig 1:**
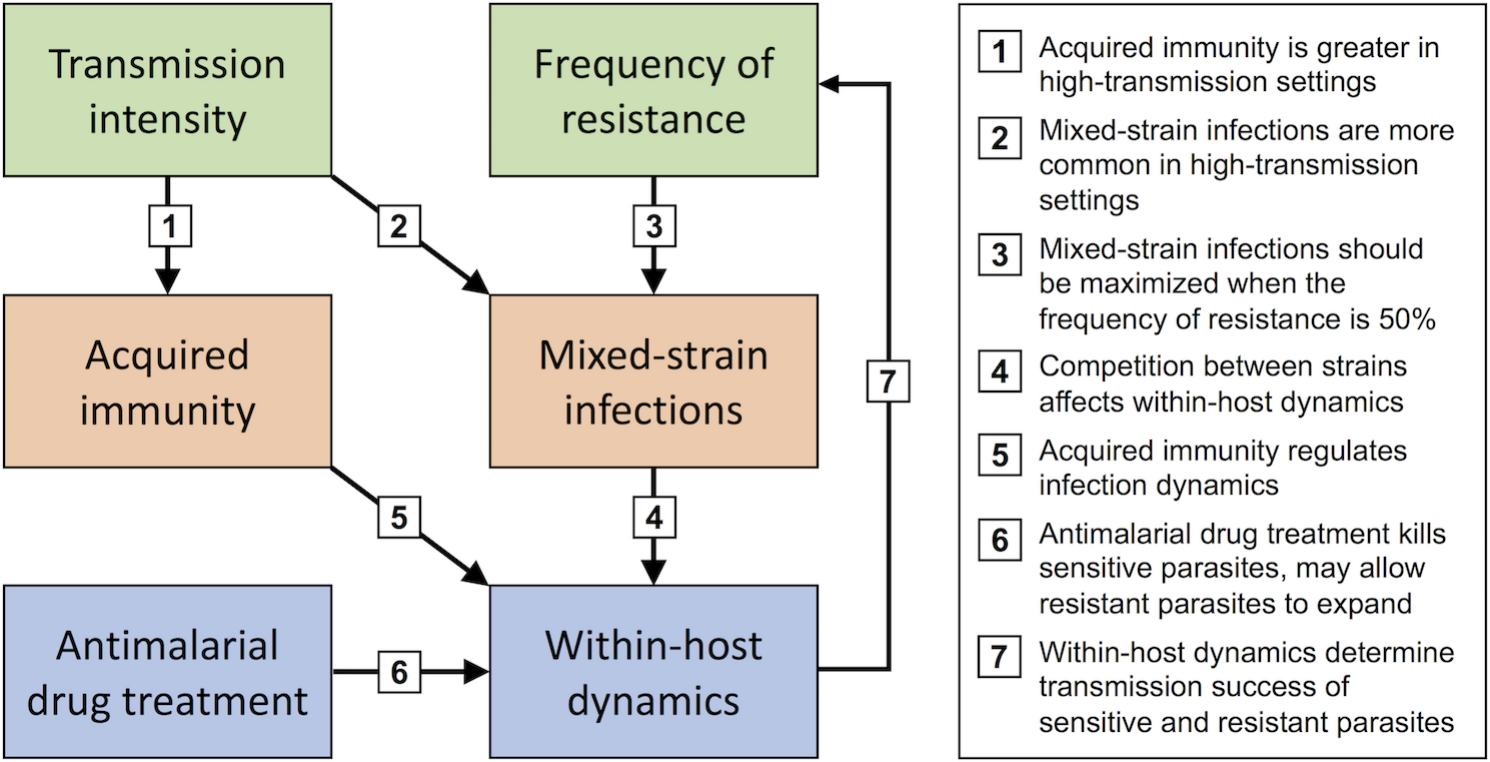
Links between transmission intensity, within-host dynamics, and the frequency of resistance in a population. Each link is numbered and briefly explained in the box to the right.

Here, we develop a fuller understanding of how within-host competition might affect the evolution of drug resistance in *P*. *falciparum*. We do so by considering the complex relationships between transmission intensity, within-host dynamics, and the frequency of drug-resistant parasites in the population (Fig 1). Transmission intensity and the frequency of resistant parasites affect within-host dynamics indirectly by determining the occurrence of mixed-strain infections as well as the acquisition of immunity; treatment with antimalarial drugs affects within-host dynamics as well. Within-host dynamics then determine the transmission potential for sensitive and resistant parasites and thereby feed directly back into the frequency of resistant parasites in the population. Thus, dynamics at the within-host and population levels are connected by “reciprocal feedbacks” that make this question amenable to using a nested model that explicitly describes dynamics on both levels [29].

In order to explore the effects of within-host competition on the establishment and spread of drug resistance, we use a fully nested, individual-based model consisting of a mechanistic model of within-host dynamics embedded into a “between-host model” that describes transmission between humans and mosquitoes. The model captures the complex network of feedbacks illustrated in Fig 1, and—unusually, even for a nested model—allows for within-host dynamics to vary as a result of ongoing exposures and acquisition of immunity. We use this model to explore key questions outlined above: whether within-host competition alone can inhibit the spread of resistance, whether competitive release can accelerate the spread of resistance, and how the net effect depends on the level of antimalarial drug use.

## Results

We developed a nested individual-based model consisting of a population of human hosts for which the within-host dynamics of parasites, red blood cells (RBCs), and immune responses are modeled using a set of ordinary differential equations (ODEs). The parasite population is assumed to comprise an effectively infinite variety of strains, which are phenotypically classified as either sensitive or resistant, and the dynamics of these two types are explicitly described in the model. (We effectively assume that there are multiple strains of each type in the population but only a single strain of each type within a given host.) Transmission between human hosts and a mosquito population is simulated using stochastic sampling algorithms; the frequency of human–mosquito contact is used as a proxy for transmission intensity.

We first give an overview of the general behavior of the within-host model and the full nested model. We illustrate the dynamics of the within-host model for a single strain and for two coinfecting strains; for the latter, we show how timing of infection, cross-reactivity between strains, and fitness differences between strains can affect within-host dynamics. For the full nested model, we show how infection prevalence, mixed-strain infections, and the gradual acquisition of immunity are affected by transmission intensity.

We then present the results of simulations in which resistant parasites are introduced at low frequency into populations in which drug-sensitive parasites are endemic. We examine the fate of the resistant parasites in low- and high-transmission settings with zero, low, or high rates of antimalarial drug treatment; we also consider how the outcomes are affected when resistant parasites suffer a fitness cost relative to sensitive parasites. In each instance, we present results for two different degrees of cross-reactivity between strains, because greater cross-reactivity tends to intensify immune-mediated competition at the within-host and population levels.

### Within-host model

**Fig 2:**
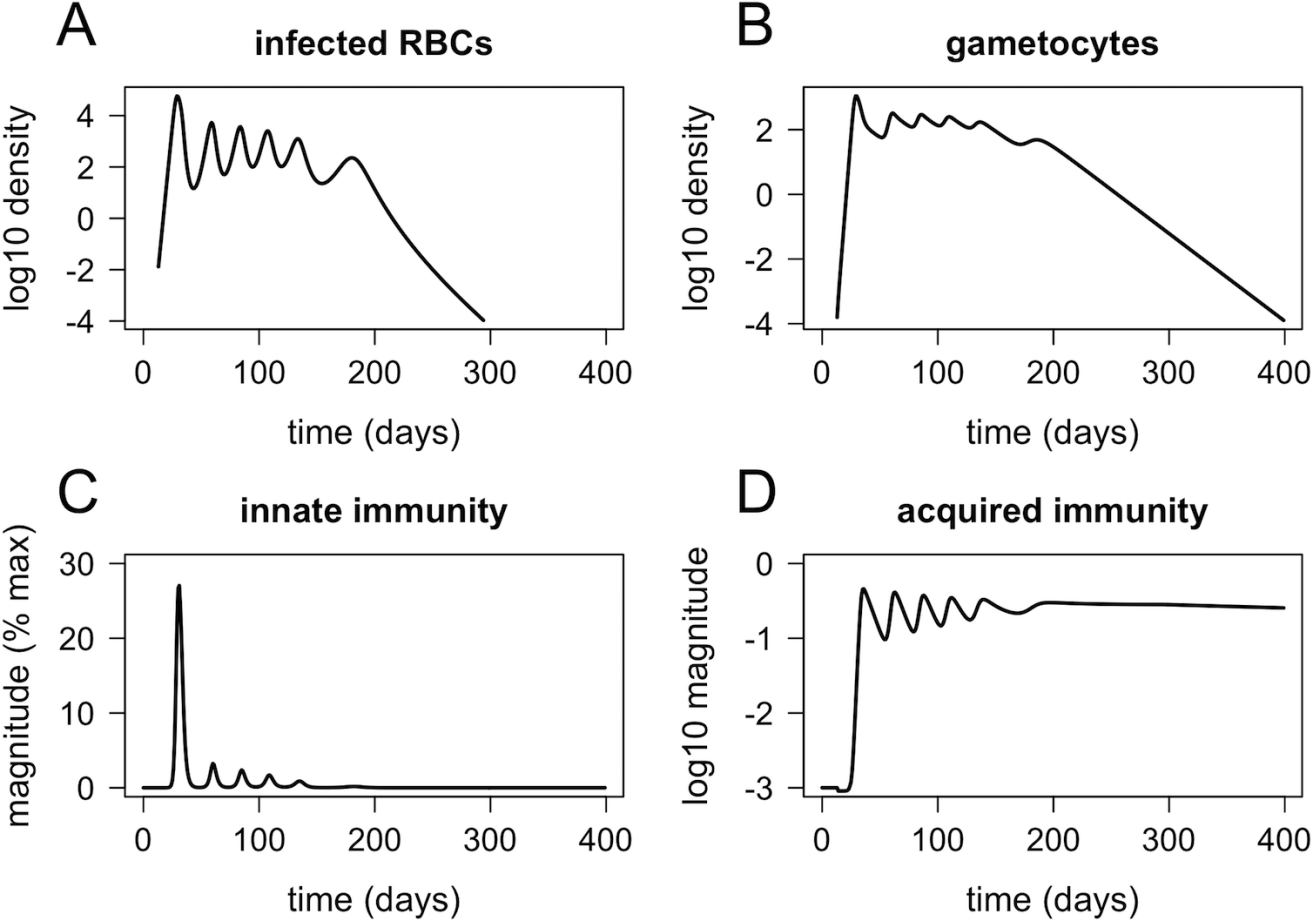
Dynamics of infected RBCs (A), gametocytes (B), innate immunity (C), and acquired immunity (D) for a naïve host (no preexisting immunity) infected with a single strain. RBC, red blood cell.

The within-host model accurately mimics the dynamics of parasites and immune responses observed in *P*. *falciparum* infections. Fig 2 shows the simulated within-host dynamics of infection with a single strain, in the absence of preexisting immunity or antimalarial drugs. The dynamics of infected RBCs (Fig 2A) are qualitatively similar to those observed in malaria-therapy patients (examples shown in [30,31]), which are still one of the best sources of data on malaria infection dynamics in naïve hosts. Gametocyte density (Fig 2B), innate immunity (Fig 2C), and acquired immunity (Fig 2D) all more or less track the dynamics of infected RBCs. Note that gametocytes persist several weeks longer than asexual parasites, consistent with observations from human malaria infections [32]. Acquired immunity fluctuates (a result of the way antigenic variation is built into the model) but eventually stabilizes at a high level that serves to reduce parasite growth in future infections.

**Fig 3:**
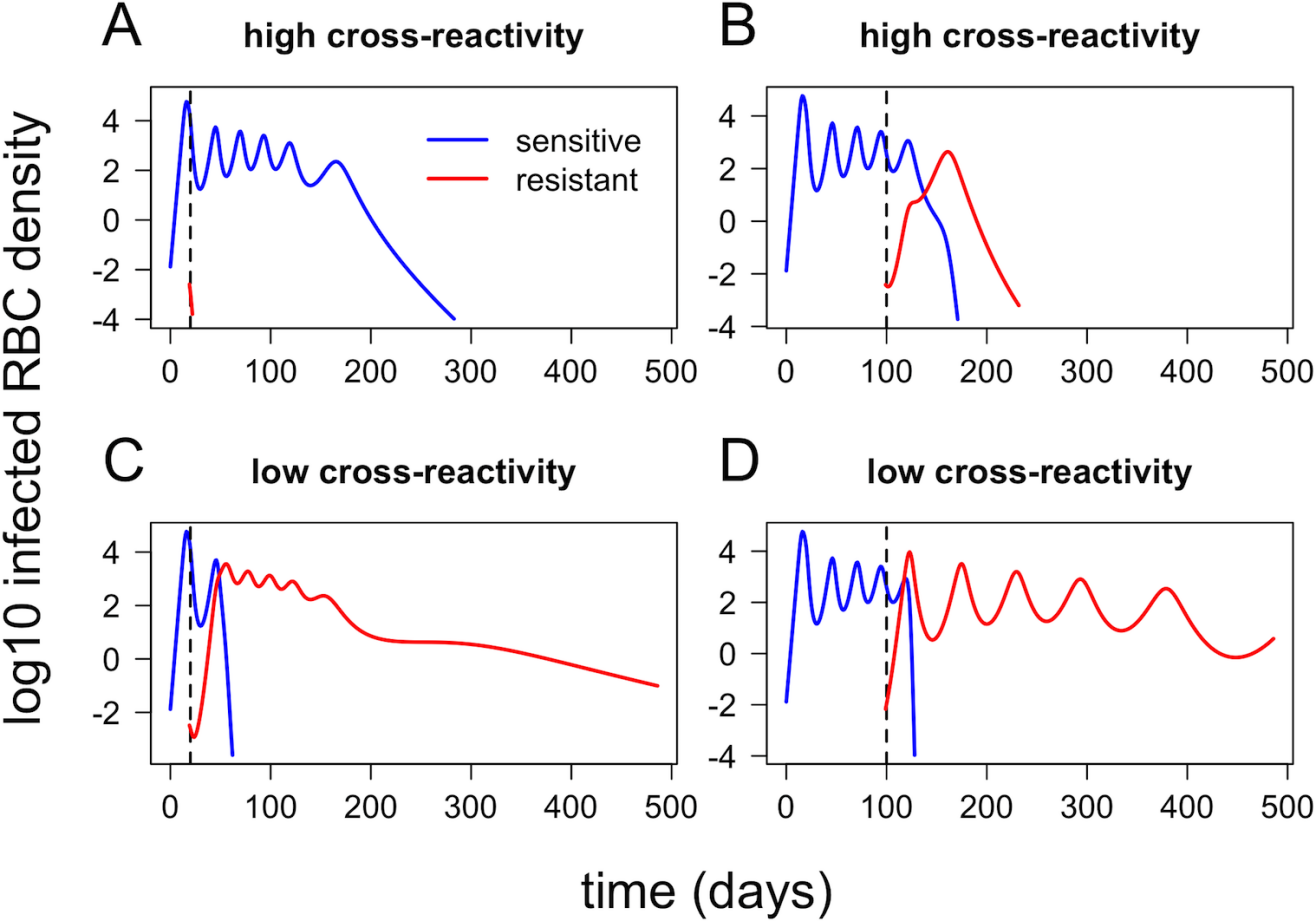
Within-host dynamics of two strains (sensitive and resistant, shown in blue and red, respectively) in a naïve host with no antimalarial drug treatment. Strains are introduced either 20 days apart (A, C) or 100 days apart (B, D), with either high cross-reactivity (A-B) or low cross-reactivity between the two strains (C-D). RBC, red blood cell.

In contrast to single-strain infections, empirical data on the dynamics of mixed-strain malaria infections in humans are extremely limited; however, two-strain dynamics produced by our within-host model are biologically reasonable and also consistent with observations from the *P*. *chabaudi* model system. Fig 3 shows within-host dynamics of two strains (sensitive and resistant), introduced either 20 or 100 days apart, with either high or low cross-reactivity between strains (cross-reactivity is controlled by antigenic overlap, the proportion of antigens or epitopes that are shared between strains). The resistant strain, which is introduced second, reaches higher densities and persists longer when cross-reactivity with the sensitive strain is small, demonstrating that cross-reactive immune responses can contribute to competition between strains. The effect of timing can be most clearly seen in the simulations with higher antigenic overlap. When the strains are introduced 20 days apart, the newly arrived resistant strain immediately goes extinct (probably due to high levels of innate immunity provoked by the drug-sensitive strain); however, when the strains are introduced 100 days apart, the resistant strain overtakes the sensitive strain because of strain-specific adaptive immune responses that disproportionately affect the sensitive parasites. This shows the sensitivity of mixed-strain infection dynamics to the timing of the introduction of different strains, a factor that has been explored in the *P*. *chabaudi* model system [19] but is frequently overlooked in mathematical models of superinfection.

**Fig 4:**
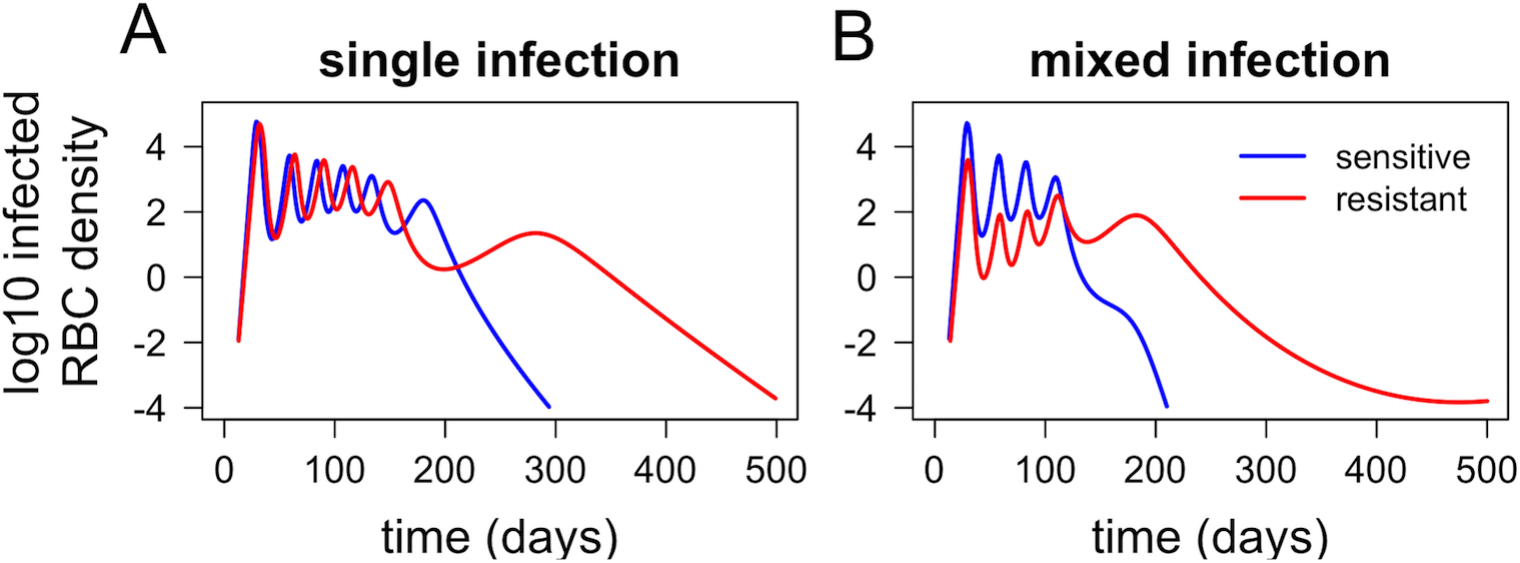
Within-host dynamics of drug-sensitive and drug-resistant strains (blue and red, respectively) in single infections (A) and in a mixed infection (B). The resistant strain is assumed to have a 10% reduction in growth rate, compared to the sensitive strain. Dynamics shown are for naïve hosts with no antimalarial drug treatment. RBC, red blood cell.

Many studies have suggested that drug resistance imposes a fitness cost, with resistant parasites exhibiting impaired growth in vivo [23,33]. When incorporating even a modest cost of resistance, our model suggests that such costs may be strongly exacerbated by within-host competition. Fig 4A shows that, in single infections (without any competition between strains), the impact of a 10% fitness cost of resistance is small; the area under the curve for the resistant strain (total parasites produced over the entire infection) is 4.5% lower than for the sensitive strain. In a mixed infection, however, the area under the curve for the resistant strain is 91.4% lower than for the sensitive strain (Fig 4B).

### Full nested model

The full nested model produces epidemiological patterns that are consistent with empirical observations. First, we show how the mosquito biting rate—the parameter that determines transmission intensity in our simulations—determines the entomological inoculation rate (EIR; the number of infective mosquito bites per person per year). EIR was estimated for simulations with four different values of *b* (mean number of mosquito bites per person per day). For simulations with *b* = 5, *b* = 10, *b* = 15, and *b* = 20, respectively, the estimated EIRs are 6.1, 25.3, 47.9, and 72.6 infective bites per person per year. These values are not fixed, since EIR depends on the total prevalence of infection in the human population, which can vary both within and between simulations (for instance, an intervention can reduce the prevalence of infection and consequently decrease EIR, even if the mosquito biting rate remains the same). The exact values are less important than the fact that for a two-fold change in biting rate, EIR more than doubles, reflecting the fact that mosquito biting rate affects not only the frequency of contact between humans and mosquitoes but also the likelihood that any particular mosquito is infective, since the prevalence of infection among humans increases with transmission intensity as well. This finding is consistent with a well-known feature of the Ross-MacDonald model, in which *R*_0_ is proportional to *a*^2^, where *a* is the rate of contact between humans and mosquitoes [34].

**Fig 5:**
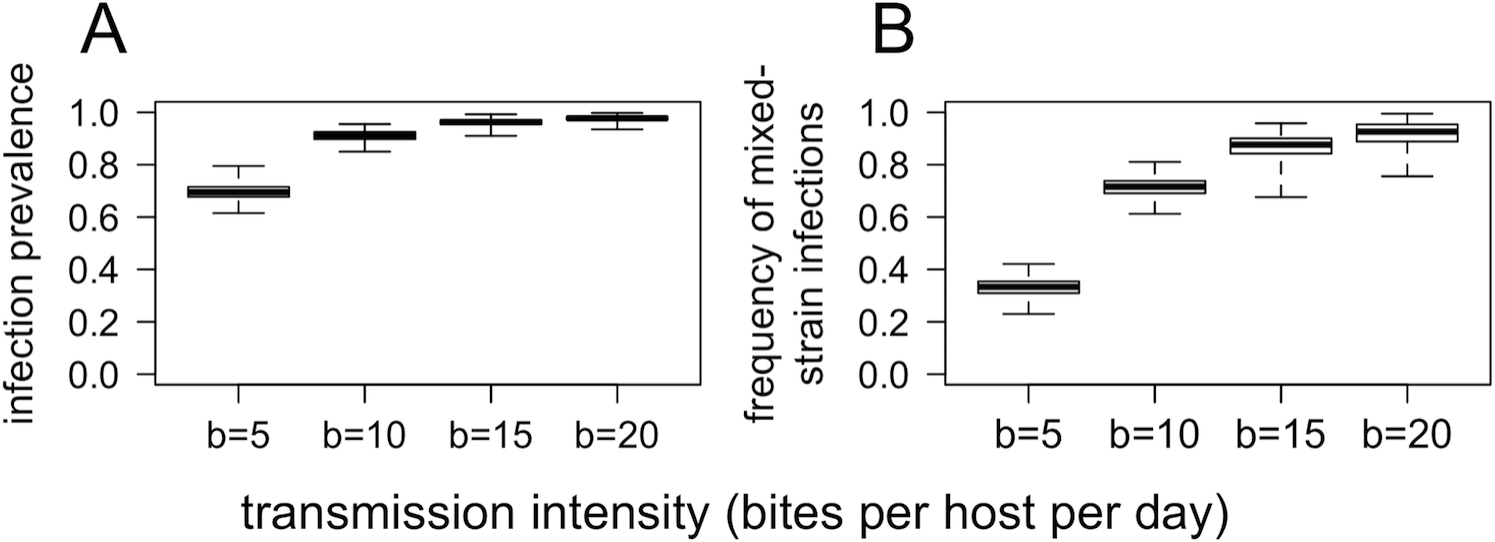
Equilibrium prevalence of infection (A) and frequency of mixed-strain infections (B) for four different transmission intensities. Data are daily measurements over a period of 2,000 days, from four independent simulations for each value of *b* (e.g., each box-and-whisker represents 8,000 point measurements). Simulations were run with no fitness differences between strains or antimalarial drug use. Box-and-whisker plots show median, interquartile range, and maximum/minimum.

Our model also reproduces the fundamental relationship between transmission intensity and prevalence of infection. Fig 5A shows the relationship between transmission intensity (determined by the mosquito biting rate, *b*) and total prevalence of infection. Prevalence increases from approximately 70% in the lowest transmission setting (*b* = 5) to near 100% in the highest transmission setting (*b* = 20). These results are consistent with epidemiological observations of the relationship between transmission intensity and infection prevalence [35]. Actual estimates of malaria prevalence are significantly lower than those given by the model; this may be attributable to the rapid host turnover in the model, as well as the limits of detection of malaria screening tools (which generally range from 1 to 100 parasites/μL).

The model also reproduces one of the key relationships outlined in Fig 1: that the frequency of mixed-strain infections increases with transmission intensity. Fig 5B shows the relationship between transmission intensity and the frequency of mixed-strain infections (in this context, meaning infections with both drug-sensitive and drug-resistant strains). The median frequency of mixed infections increases from 33% for the lowest transmission intensity (*b* = 5) to 93% for the highest transmission intensity (*b* = 20). These results are consistent with observed correlations between transmission intensity and average multiplicity of infection (number of strains per host) [17].

**Fig 6:**
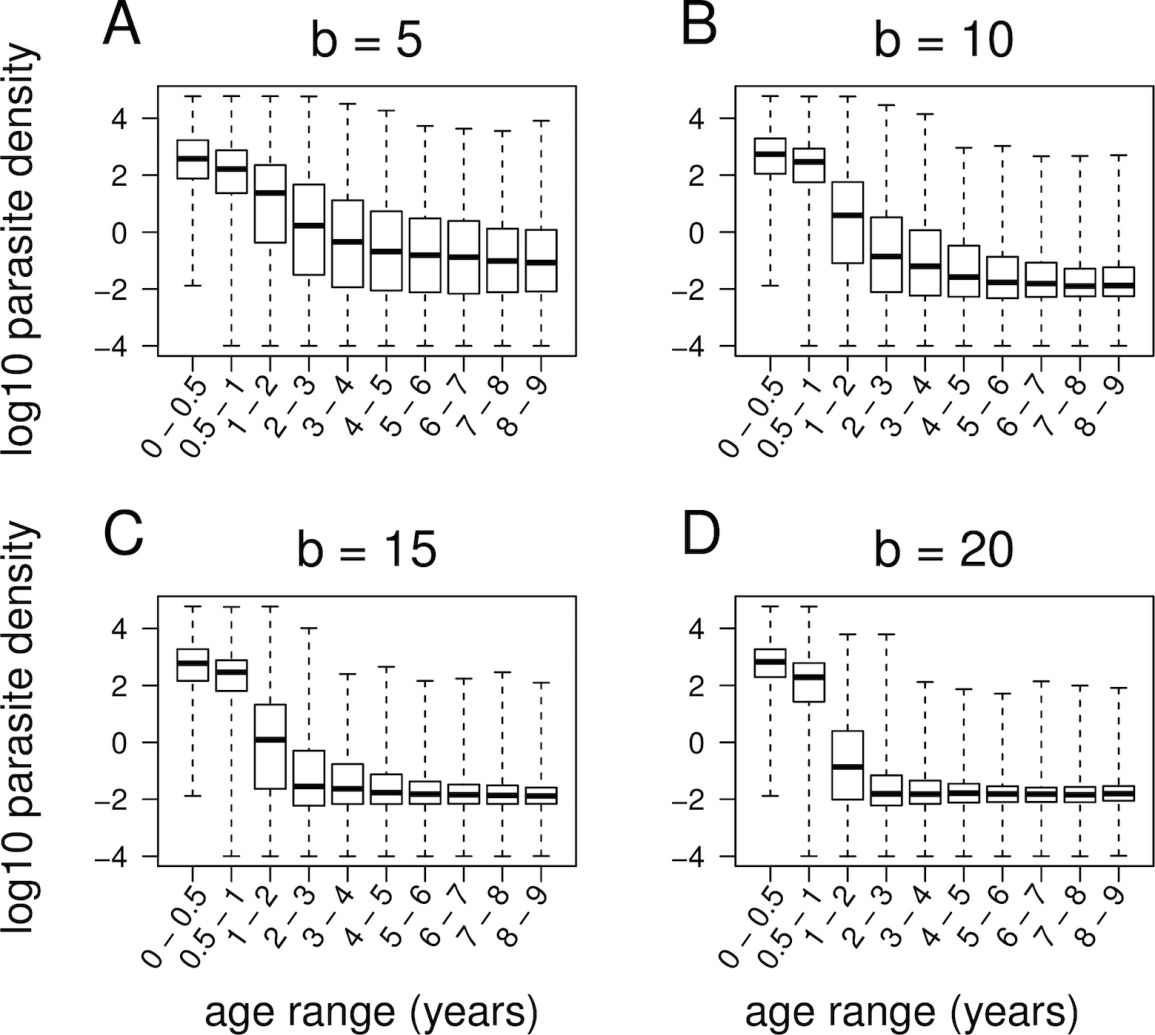
Equilibrium distribution of parasite densities (log_10_ transformed) for age groups ranging from 0 to 9 years, for four different levels of transmission intensity (*b* = number of mosquito bites per person per day). Data are daily measurements over a period of 2,000 days. Simulations assumed no antimalarial drug use or fitness differences between strains. Box-and-whisker plots show median, interquartile range, and maximum/minimum.

Epidemiological studies have observed that immunity to malaria is acquired over time, with immunity developing more rapidly as transmission intensity increases; in high-transmission settings, clinical immunity to malaria is more or less fully developed by five years of age [13,36]. Again, our model accurately reproduces these patterns. Fig 6 shows the relationship between age and parasite density for four different levels of transmission intensity. In all settings, parasite density decreases with age, but this decrease is more rapid in higher transmission settings, consistent with empirical observations.

### Simulation results

In order to understand how within-host competition affects the spread of resistant parasites at the population level, it is helpful to first examine what happens in the absence of selection. We ran simulations lasting 8,000 days (21.9 years), with sensitive parasites introduced at the start and resistant parasites introduced after 3,000 days (8.2 years). Resistant parasites were randomly introduced into 2% of human hosts, with no antimalarial drug use and no fitness cost of resistance. We ran sets of 10 independent simulations for four conditions: low transmission (*b* = 5) and high transmission (*b* = 15) with high cross-reactivity and low cross-reactivity between strains. As discussed above, greater cross-reactivity tends to intensify competition between strains.

**Fig 7:**
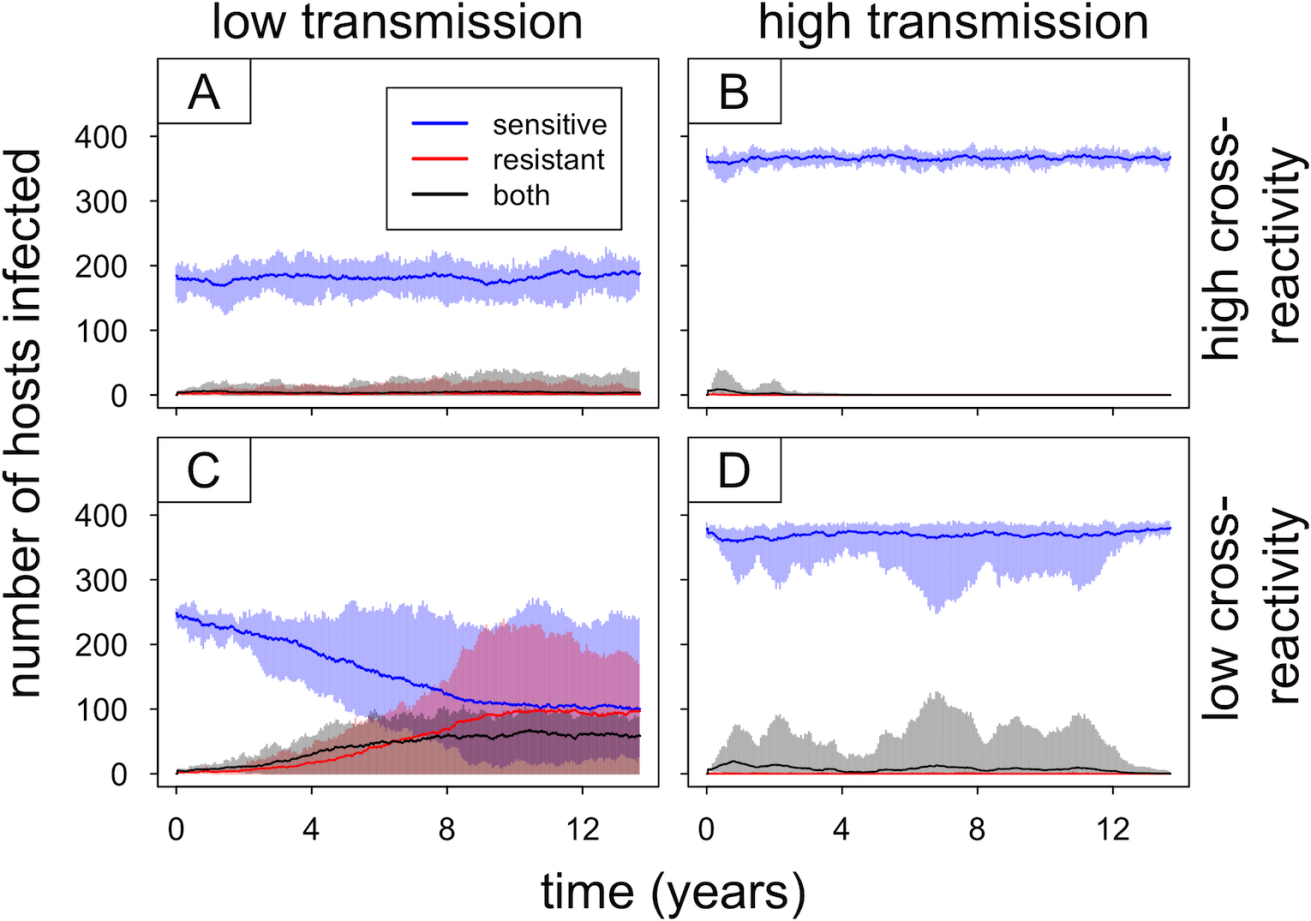
Introduction of drug-resistant parasites in the absence of fitness costs or antimalarial drug use. Results are shown for low-transmission (*b* = 5) and high-transmission (*b* = 15) settings with high or low cross-reactivity between strains. Each panel summarizes 10 independent simulations with identical parameters and starting conditions, with solid lines showing mean values and shaded areas depicting the range of observed values (blue, red, and black representing infections with sensitive, resistant, or both types of parasites, respectively). The drug-resistant parasites were introduced into 2% of the human host population 3,000 days after the introduction of sensitive parasites (dynamics prior to introduction of resistant parasites not shown).

As shown in Fig 7, the resistant parasites are able to persist longer and achieve higher prevalence in low-transmission settings, particularly with low cross-reactivity between strains. The simulations continue for 5,000 days (13.7 years) after introducing the resistant parasites; the frequency with which the resistant parasites go extinct in this time frame differs across the four conditions tested. The resistant parasites go extinct more often in high-transmission settings than in low-transmission settings (10 out 10 simulations for high transmission/high cross-reactivity compared to 8 out of 10 for low transmission/high cross-reactivity, and 7 out of 10 simulations for high transmission/low cross-reactivity compared to 2 out of 10 for low transmission/low cross-reactivity). The length of time that the resistant parasites persist prior to going extinct also varies: looking only at the cases in which the resistant parasites go extinct, the mean time to extinction is lower for high-transmission settings than for low-transmission settings (2.2 years for high transmission/high cross-reactivity compared to 5.8 years for low transmission/high cross-reactivity, and 2.3 years for high transmission/low cross-reactivity compared to 3.3 years for low transmission/low cross-reactivity).

The cumulative prevalence of the resistant parasites is also starkly different across the four conditions tested. The total number of infection days for resistant parasites is an order of magnitude greater in low-transmission settings compared to high-transmission settings (Table 1). These differences in cumulative prevalence—a measure that accounts for factors such as duration of infection, as well as force of infection and time to extinction—suggest that low-transmission settings are more conducive to persistence and transmission of newly introduced resistant parasites and may consequently offer more opportunities for the rare resistant parasites to be selected by antimalarial treatment. In contrast, persistence of the resistant parasites appears to be strongly inhibited in high-transmission settings because of within-host competition from sensitive parasites occupying the majority of hosts. Cumulative prevalence of the resistant strain is also an order of magnitude lower in the simulations with high cross-reactivity between strains, compared to those with low cross-reactivity. This suggests that immune-mediated competition, which is stronger when cross-reactivity between strains is greater, might play an important role in limiting the persistence of newly introduced resistant parasites.

**Table 1:** Cumulative prevalence (total infection days) for resistant parasites in simulations with no antimalarial drug treatment.

Cross-reactivity	Transmission intensity	Total infection days for resistant parasites
High	Low	3.1×10^4^ (range: 1.6×10^3^ − 1.9×10^5^)
	High	4.0×10^3^ (range: 1.0×10^3^ − 1.1×10^4^)
Low	Low	4.7×10^5^ (range: 4.1×10^3^ − 7.9×10^5^)
	High	3.7×10^4^ (range: 9.6×10^2^ − 2.9×10^5^)

**Fig 8:**
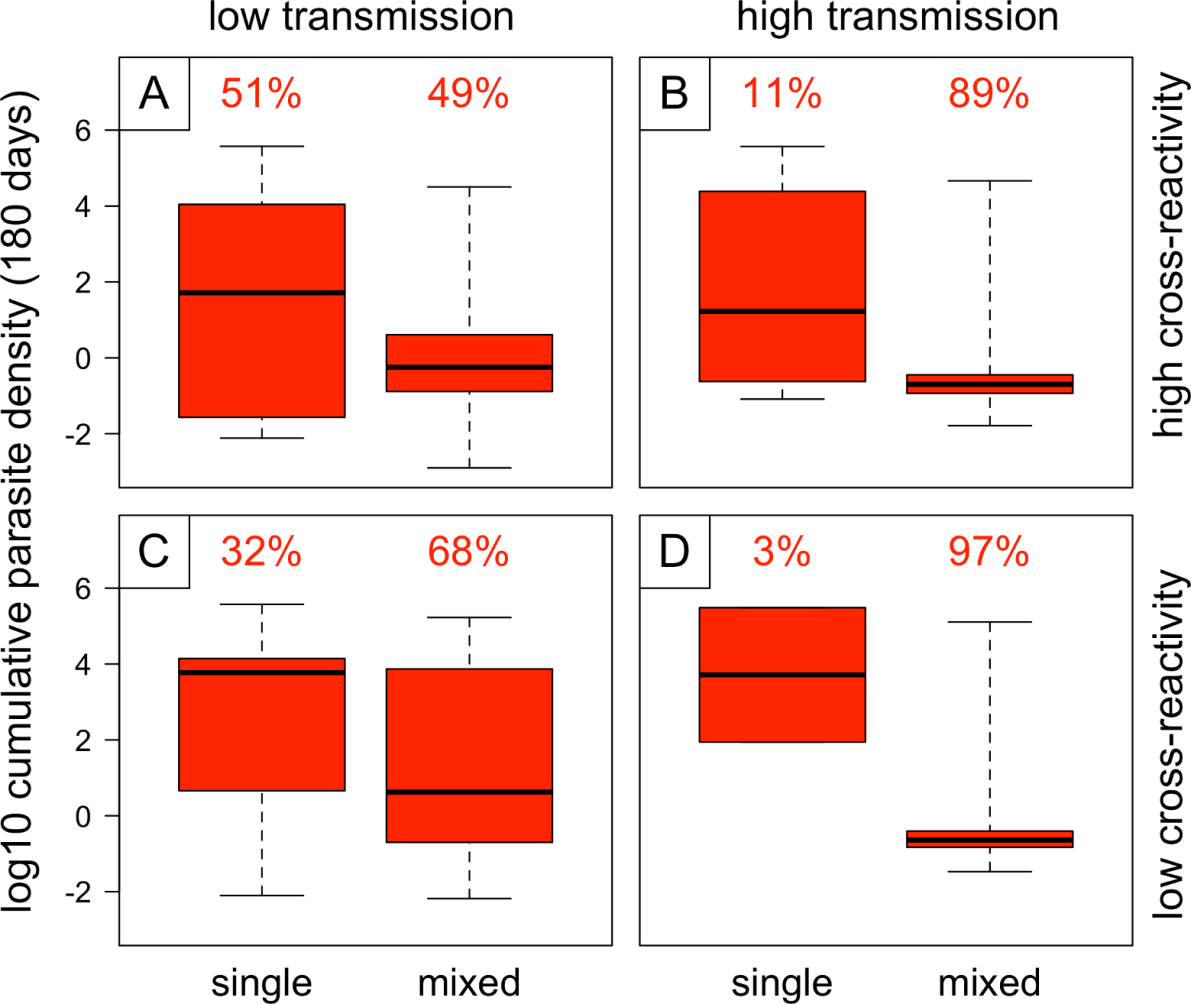
Cumulative densities (over 180 days) of resistant parasites in the 2% of hosts initially infected, stratified according to whether hosts are infected with a sensitive strain at the time the resistant strain is introduced (“single” and “mixed” indicating the absence and presence, respectively, of drug-sensitive parasites in the same host). The proportions of infections falling into each category (single and mixed) are shown above the box-and-whisker plots. Results are shown for low transmission (*b* = 5) and high transmission (*b* = 15) with either high cross-reactivity or low cross-reactivity between strains, and 10 independent simulations for each set of conditions; simulations assumed no antimalarial drug use or fitness cost of resistance.

The higher probability of extinction of resistant parasites in high-transmission settings appears to be driven by more frequent and more severe within-host competition between sensitive and resistant strains. Fig 8 shows cumulative densities of resistant parasites in hosts that are uninfected when the resistant parasites are introduced (single infections) and in hosts that already harbor sensitive parasites when the resistant parasites are added (mixed infections); the proportion of infections falling into each category (single or mixed) is shown above each box-and-whisker plot. Mixed infections occur much more frequently in high-transmission settings, meaning the resistant parasites encounter competitive suppression more often than in the low-transmission settings. Unsurprisingly, cumulative densities of the resistant parasites are reduced in mixed infections compared to single infections; interestingly, the reduction is more severe in high-transmission settings, suggesting that the higher average level of acquired immunity in the high-transmission conditions may contribute to competitive suppression of the newly introduced resistant parasites.

**Fig 9:**
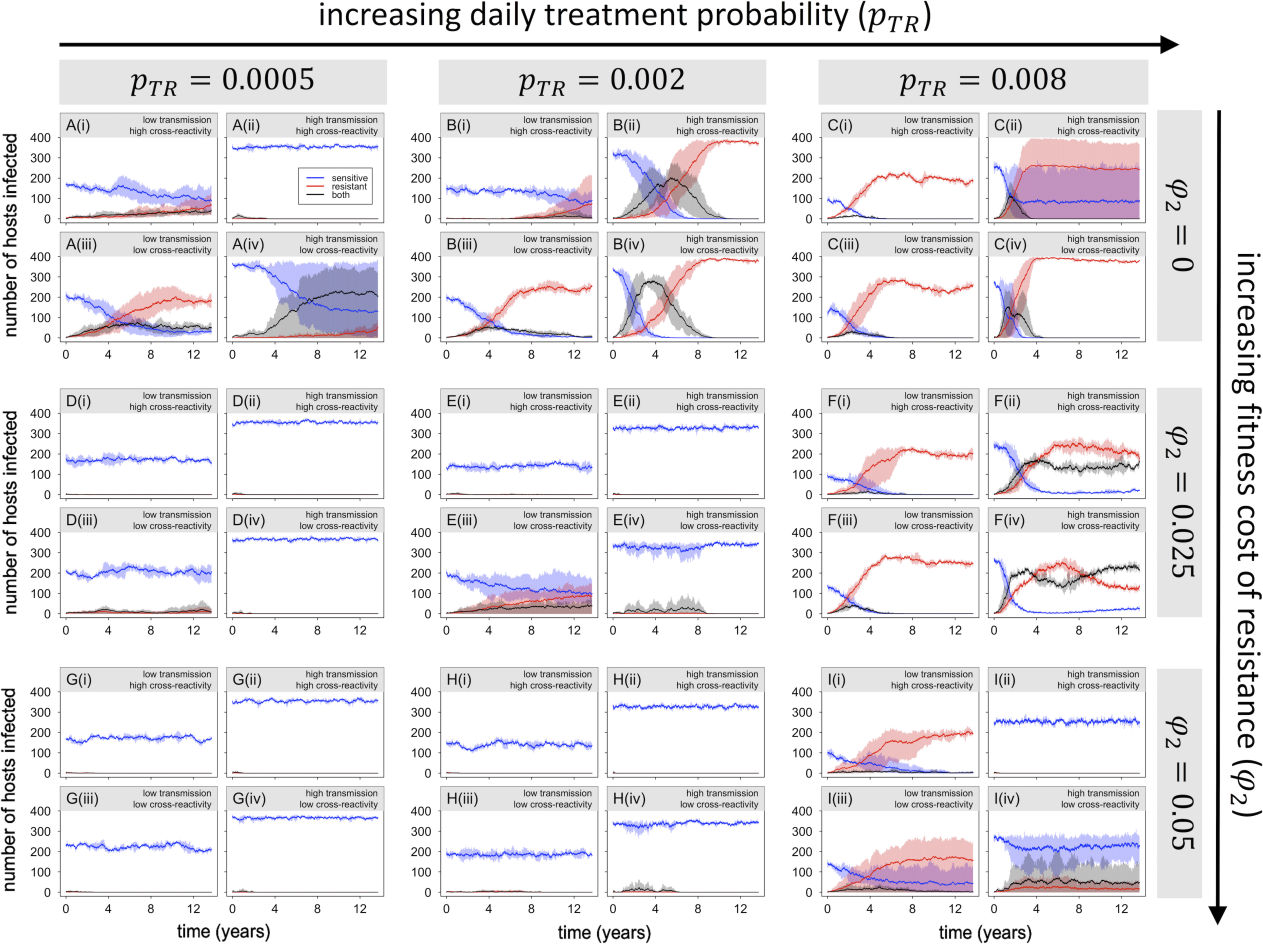
Introduction of resistant parasites into populations with varying levels of antimalarial drug use and fitness costs of resistance. Panels A–I vary in treatment rate (daily probability of infected host starting treatment) and fitness cost (proportional reduction in within-host growth rate for the resistant type). As in Figs 7 and 8, each four-part panel presents results for (i) low transmission/high cross-reactivity, (ii) high transmission/high cross-reactivity, (iii) low transmission/low cross-reactivity, and (iv) high transmission/low cross-reactivity. For all figures, solid lines and shaded areas show mean and range of 3 independent simulations (blue, red, and black representing infections with sensitive, resistant, or both types of parasites, respectively). Note that in some panels, such as C(ii), shading for one or more colors covers a wide area; this is generally caused by the resistant strain going extinct in some but not all of the replicate simulations.

The above analyses demonstrate that within-host competition can inhibit the spread of resistance in the absence of selection from antimalarial drugs or a fitness cost of drug resistance. The next step is to consider how within-host competition might affect the spread of resistance under selection. Antimalarial drug treatment and fitness costs of resistance, of course, exert opposing selective pressures on resistance; the net outcome will depend on the relative strengths of these selective forces. Fig 9 shows the results of simulations with three different rates of antimalarial drug use combined with three different fitness costs (one of which is zero, in order to look at the effect of selection from antimalarial drugs alone). Results for a slightly expanded range of parameter values are presented in S1 Fig.

Panels A–C of Fig 9 show the results of simulations with no cost of resistance and varying levels of antimalarial drug use. With the lowest rate of treatment (Fig 9A), the results are fairly similar to those with no selection (Fig 7); in particular, the resistant parasites consistently go extinct in the high-transmission/high-cross-reactivity setting, despite increasing in frequency in the low-transmission/high-cross-reactivity setting. This suggests that within-host competition is able to prevent the establishment of resistance in high-transmission settings even in the presence of positive selection. With higher rates of treatment (Fig 9B and 9C), however, resistance is able to spread in all settings, but the relationship between transmission intensity and spread of resistance flips, such that the rate of spread of resistance is higher in high-transmission settings. Taken together, these results suggest that in high-transmission settings, drug-resistant parasites are at higher risk of extinction when rare but may actually spread more rapidly if selection is sufficiently strong to avoid extinction in the early stages.

Panels D–I of Fig 9 show results of simulations that include fitness costs of drug resistance as well as antimalarial drug use. These yield three main observations. First, with a low rate of treatment and/or a high fitness cost of resistance, the establishment of resistant parasites is again inhibited in high-transmission settings (Fig 9D, 9E, 9G and 9H). Second, with a very high rate of treatment, resistance spreads to fixation in both low- and high-transmission settings, even in the presence of a fitness cost (S1D, S1H, S1L and S1Q Fig). Third, with treatment rate and fitness cost both at intermediate levels, resistance is able to spread in both low- and high-transmission settings but often fails to reach fixation (Fig 9F and 9I); this effect is particularly pronounced in high-transmission settings. The stable coexistence of sensitive and resistant parasites may result, in part, from exacerbation of the fitness cost of resistance in mixed-strain infections (as shown in Fig 4), which will make the average fitness of the resistant parasites depend on the frequency of mixed-strain infections, as well as the rate of antimalarial drug use.

A key result from these simulations is the fact that drug resistance often spreads (increases in frequency) more quickly in high-transmission settings, despite being at higher risk of extinction when rare. This could be partly attributable to the fact that the prevalence of infection is greater in high-transmission settings; thus, if equal proportions of infections are treated in low- and high-transmission settings, a higher proportion of hosts will be treated (and therefore effectively unavailable to drug-sensitive parasites) in high-transmission settings. However, the spread of resistance in high-transmission settings may also be accelerated by “competitive release,” which refers to enhanced growth of the resistant parasite population in a host following removal of drug-sensitive competitors by drug treatment.

**Fig 10:**
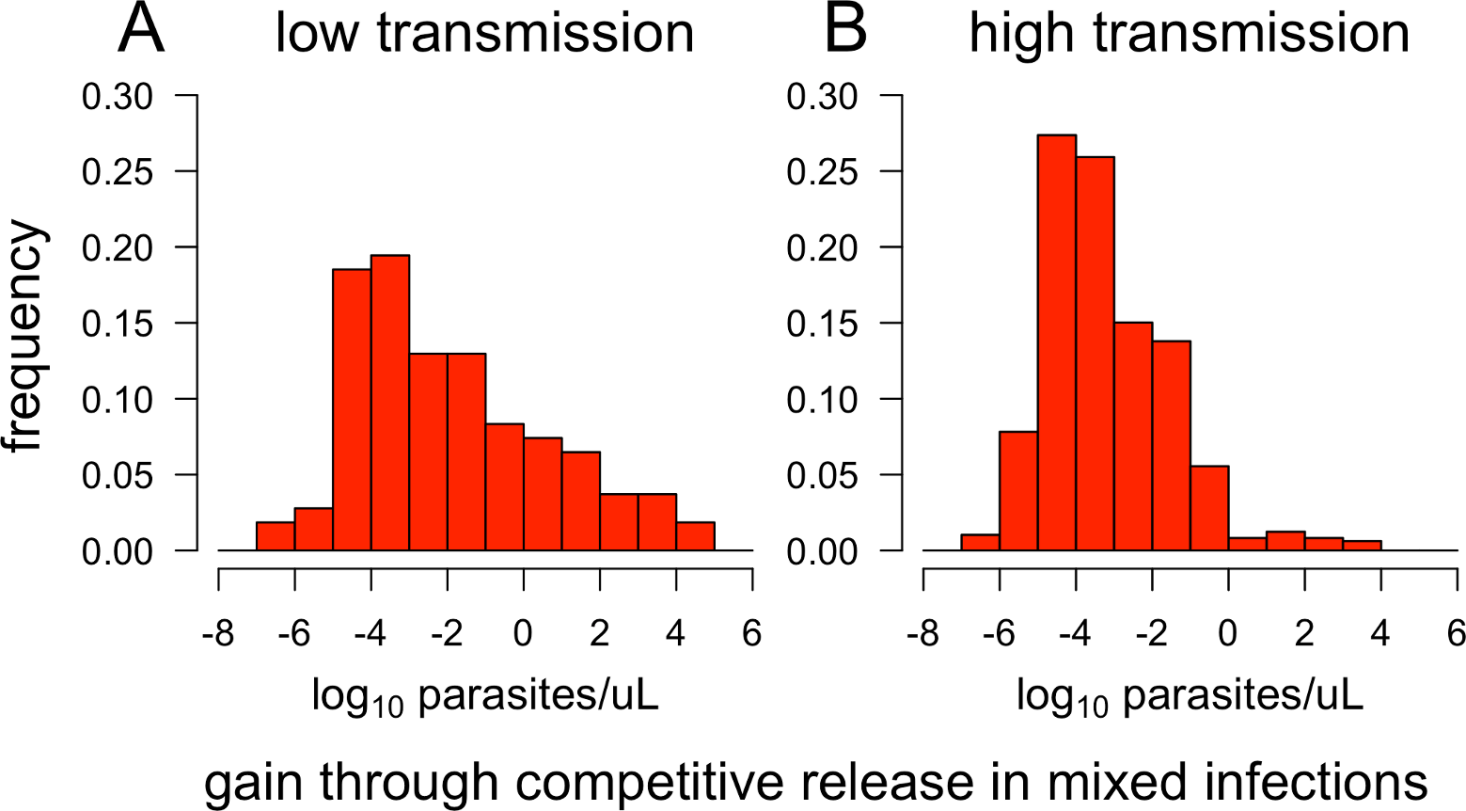
Net gains achieved by resistant parasites following antimalarial drug treatment of mixed (sensitive + resistant) infections. Using results from the simulations shown in Fig 9B(i-ii), we compared the densities of resistant parasites at the end of every 14-day treatment window to the densities that would be expected if the same infections had continued untreated for that 14-day period. For each treatment instance, the net gain is reported as the density of resistant parasites after 14 days of treatment minus the density expected after 14 days without treatment.

Evidence for competitive release at the within-host level can be seen by comparing the posttreatment density of resistant parasites to the density that would have been observed if the host had gone untreated. We examined the within-host dynamics underlying spread of resistance in low- and high-transmission settings, using model output for one simulation from Fig 9B(i) and one from Fig 9B(ii). Fig 10 shows the distributions of resistant “gains” through competitive release in treated mixed infections from each of these simulations. In most cases, the net gains for resistant parasites are small: 77% of treated mixed infections in low-transmission settings and 97% in high-transmission settings show net gains of less than 1 parasite/μL. However, a minority of treated mixed infections (approximately 9% in the low-transmission setting and 1% in the high-transmission setting) show net gains of over 100 parasites/μL.

In high-transmission settings, the gains achieved by resistant parasites through competitive release might be somewhat reduced due to higher levels of acquired immunity (which acts in a more strain-specific manner than resources or innate immunity and is also slower to respond to changes in parasite density). However, this decreased competitive release is likely offset somewhat by the greater frequency of mixed-strain infections (roughly 28% of all infections in the high-transmission settings—counting only over the period during which sensitive and resistant coexist in the population—versus 8% in the low-transmission setting). In addition, the nonlinear relationship between gametocyte density and transmission to mosquitoes means that the increase in fitness obtained from competitive release is not necessarily proportional to the change in parasite density.

## Discussion

One of the most compelling pieces of evidence that within-host competition could inhibit the evolution of resistance comes from the results of simulations with no selection at all, in which the only forces at work are ecological ones. Simulations with no antimalarial drug use and no intrinsic fitness differences between sensitive and resistant parasites show that resistant parasites introduced at low frequency persist longer in a low-transmission setting than in a high-transmission setting with otherwise identical parameters. Analysis of the within-host dynamics in these simulations shows that, in a high-transmission setting, the newly introduced resistant parasites almost always finds themselves in mixed-strain infections, in which they suffer from competition with drug-sensitive parasites. As the “newcomers” in these mixed infections, the resistant parasites suffer disproportionately from within-host competition, even though they are not intrinsically less fit; this effect of prior residency has been observed in the *P*. *chabaudi* model system [19]. In contrast, in low-transmission settings, the newly introduced resistant parasites are more likely to find “unoccupied territory” in hosts without drug-sensitive competitors. As a result, the resistant parasites suffer less from within-host competition and are better able to persist in the population. Since the majority of *P*. *falciparum* infections are probably untreated at any given time [16], being able to persist in the population long enough to experience episodes of positive selection by antimalarial drugs is likely to be a key determinant of the evolution of resistance.

These results suggest that within-host competition may impede the spread of resistance even in the absence of a fitness cost of resistance. Other theoretical studies have concluded that within-host competition would slow the evolution of resistance in high-transmission settings, but these results are contingent on a fitness cost of resistance being exacerbated in mixed-strain infections [25,26]. Results from simulations with a fitness cost of resistance are consistent with the idea that costs may be exacerbated in mixed infections, and this may well contribute to the impaired spread of resistance in high-transmission settings; however, fitness costs do not appear to be intrinsic to the effects of within-host competition on the spread of resistance. However, if the magnification of fitness costs in mixed-strain infections serves to prevent the fixation of resistance in the parasite population, the resulting preservation of drug-sensitive parasites could play a key role in the recovery of drug susceptibility when an antimalarial drug is retired—a phenomenon that has been documented in numerous areas [23,37–39] and that has raised hopes for a viable “drug cycling” strategy to manage resistance in *P*. *falciparum*.

Across all simulations, the resistant parasites persist longer and/or spread more rapidly with lower cross-reactivity between strains. Cross-reactivity, in this context, determines the extent to which resistant parasites are affected by acquired immunity to sensitive parasites, and vice versa; greater cross-reactivity therefore translates to stronger immune-mediated “apparent competition.” The results showing that lower cross-reactivity is favorable for newly introduced resistant parasites are consistent with the idea that within-host competition may inhibit the spread of resistance. Furthermore, cross-reactivity is likely to be greatest for sensitive and resistant strains drawn from the same local parasite population; a resistant strain imported from a distant population may have markedly lower cross-reactivity with local drug-sensitive strains, giving it a competitive advantage over local parasites [40]. This may favor the establishment of imported resistant parasites in the population and facilitate their spread; this is an interesting hypothesis to explain the spread of drug-resistant genotypes from Southeast Asia across much of sub-Saharan Africa [4,5].

Simulations with antimalarial drug use also show resistance emerging more readily in low-transmission settings—more readily, but not necessarily more quickly. In cases in which the resistant parasites are able to avoid extinction, resistance actually spreads more rapidly in high-transmission settings than in low-transmission settings with otherwise identical parameters. There are two mechanisms that may contribute to this rapid spread of resistance in high-transmission settings. The first is simply that the higher prevalence of infection in high-transmission settings translates into a higher overall rate of use of antimalarial drugs, effectively increasing the strength of selection for resistance. The second is that treatment of mixed-strain infections results in “competitive release” of drug-resistant parasites; this occurs in both low- and high-transmission settings but more frequently in the latter because of higher frequencies of mixed-strain infections. In contrast to prior experimental and modeling studies [9,18,20], we find that the gains attained through competitive release are generally modest; with weaker drug-resistant phenotypes (such as delayed clearance in response to artemisinin-based therapies), the gains are likely to be even smaller. However, with drugs that target gametocytes as well as asexual parasites, treatment of mixed infections may enhance transmission of resistant parasites even without large gains from competitive release, since rapid clearance of drug-sensitive gametocytes will reduce competition for transmission to mosquitoes. Thus, the impact of competitive release is likely to depend on the mode of action and degree of resistance to the drug(s) being used.

Overall, the results presented here suggest that the relationship between transmission intensity and evolution of resistance may be more nuanced than previously appreciated. This nuance arises in the distinction between establishment of resistance and spread of resistance [41,42]. High-transmission settings may be less conducive to the establishment of resistance, since the probability of extinction for newly introduced resistant parasites is higher [43]. However, the rate of spread of resistance can nevertheless be greater in high-transmission settings, as a result of competitive release, a higher overall frequency of antimalarial drug use, or both. These opposing findings help to reconcile previous models predicting rapid evolution of resistance in high-transmission settings [9,28] with the observed tendency for resistance to evolve in low-transmission settings. The distinction between probability of establishment and rate of spread may be a fruitful one to explore further in models of drug resistance evolution. For instance, although models have previously been used to explore the effects of recombination on the spread of multilocus drug resistance, the impact of recombination on establishment of multiple-mutant genotypes in the population, particularly in the presence of epistatic interactions, may be an interesting puzzle to explore in future work.

Finally, we emphasize that this model cannot rule out the possibility that recombination, unequal rates of antimalarial drug use, and/or fitness costs of resistance may be important drivers of the observed relationship between transmission intensity and evolution of drug resistance. However, our results show that the observed relationship can be qualitatively explained without invoking any of the three. A key area in which to expand this work is consideration of how these different factors might interact—for example, how the effects of within-host competition might change when mutations at multiple loci contribute to resistance. A great deal of theoretical and empirical work is still needed to quantify the relative contributions of recombination, selection pressure, within-host competition, and other mechanisms to variation in the rate of drug resistance evolution in *P*. *falciparum*.

Understanding the relationship between transmission intensity and evolution of resistance is likely to lead to improved strategies for controlling malaria and managing drug resistance [43]. For instance, the results presented here suggest that the fate of drug-resistant parasites may be more sensitive to the rate of antimalarial drug use in high-transmission settings, in the sense that strong selection might be a prerequisite for drug-resistant parasites to spread at all in these settings. Thus, interventions such as mass drug administration (MDA) may present different risks in low- versus high-transmission settings; although increased use of antimalarial drugs will always increase selection for resistance, in low-transmission settings, this seems likely to be a matter of degree, whereas in high-transmission settings, increased selection has the potential to “tip the scales” in favor of resistance, allowing resistant parasites to establish and spread where they otherwise would not. (It should be noted, however, that MDA is primarily recommended for use in low-transmission settings in pursuit of malaria elimination.) More broadly, reductions in malaria transmission could—paradoxically—increase opportunities for resistance to evolve, necessitating increased vigilance and/or compensatory measures to ensure that resistance does not gain a foothold in newly “unoccupied territory” [44,45]. Knowledge of the factors that limit—or drive—the spread of resistance will aid in optimizing control strategies for a wide range of different endemic settings [46,47].

## Materials and methods

**Fig 11:**
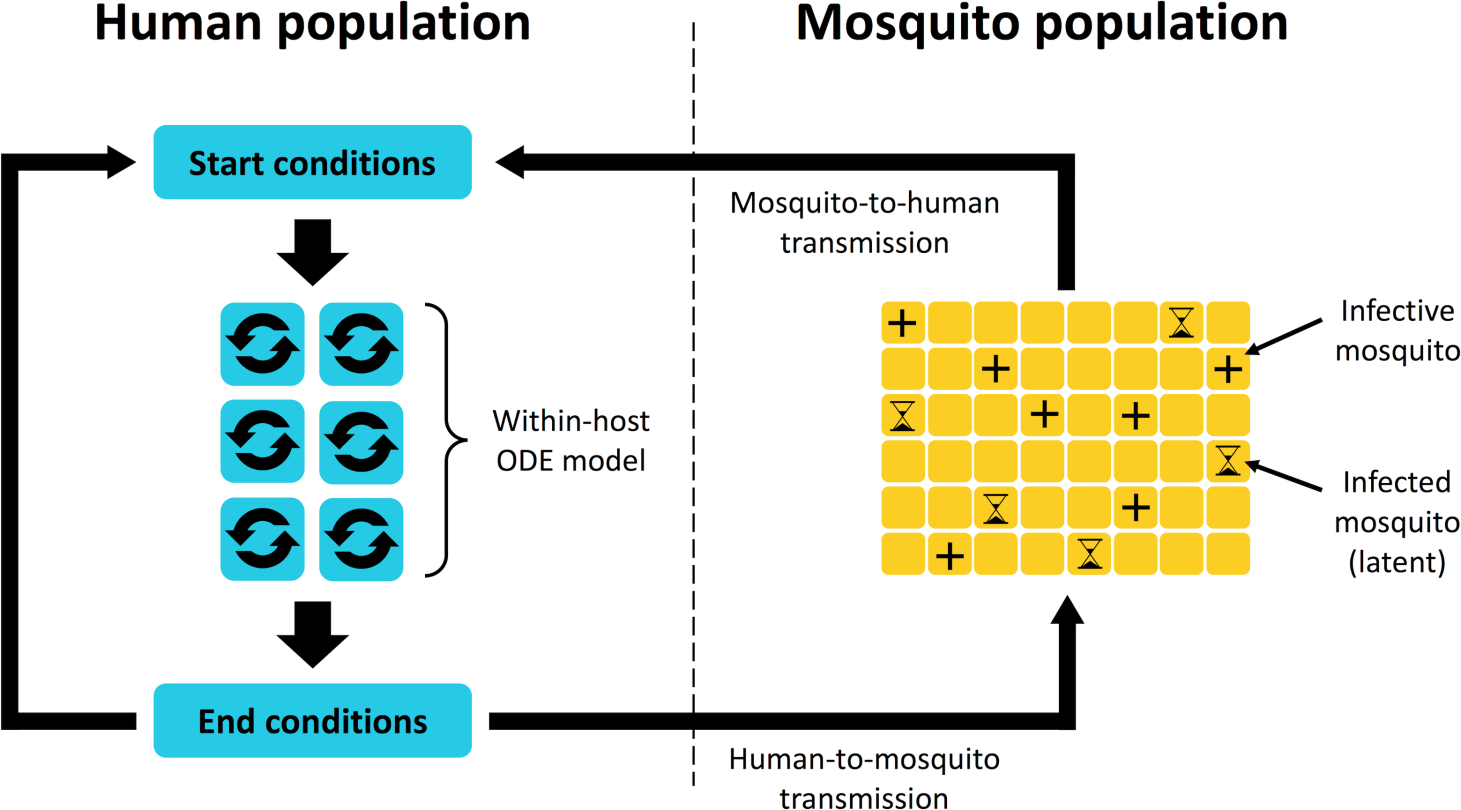
Overall structure of the full nested model. Parasites are continually transmitted from humans to mosquitoes and vice versa; dynamics of infections in humans are modeled using a system of ODEs, while infections in mosquitoes are tracked as they progress through a latent period and become infective. The within-host model tracks infection dynamics over 24-hour intervals punctuated by daily “contact” between humans and mosquitoes, during which transmission of parasites occurs. Once parasites are transmitted from a mosquito to a human, their population dynamics are governed by the within-host model. ODE, ordinary differential equation.

We use a nested model of parasite population dynamics: a model of within-host dynamics embedded into another model that simulates parasite transmission between humans and mosquitoes. Essentially, the model tracks a population of humans, a population of mosquitoes, and the parasites that circulate among them. Infections in mosquitoes are tracked over time, while the dynamics of infection in each human host are modeled using ODEs. The model simulates within-host dynamics in 24-hour increments interspersed with daily transmission of parasites between humans and mosquitoes; any new parasites that are introduced to a human host are incorporated into the population of parasites tracked by the within-host model. The general structure of the full model is illustrated in Fig 11.

The model is designed to explore questions related to the evolution of drug resistance; therefore, it actually describes the dynamics of two “types” of parasite: drug sensitive and drug resistant. We assume the parasite population is comprised of a variety of strains, which are phenotypically classified as either sensitive or resistant (in this model, resistance is absolute; we do not consider multiple resistance mutations or different degrees of resistance—such as delayed-clearance phenotypes as seen with artemisinin resistance—but doing so will be an important direction for future work). Different strains are assumed to have partial overlap in their antigens; for computational tractability, we assume that any two strains have the same degree of overlap, and the number of strains in the population is infinite. Within an individual host, all of the parasites of one type are considered to constitute a single strain; thus, an infection with only one type is a “single-strain infection,” while one with both types is a “mixed-strain infection”.

We now give an overview of the within-host model, the between-host (transmission) model, and a few other important aspects. A detailed description of the model is provided in S1 Text.

### Within-host model

The within-host model consists of a system of ODEs that describe the dynamics of infection for two parasite “types” (sensitive and resistant, denoted with subscripts 1 and 2, respectively). The dynamics of the following components are described:

- Uninfected RBCs (*X*)
- Infected RBCs of each type (*Y*_1_,*Y*_2_)
- Merozoites (extracellular parasites) of each type (*S*_1_,*S*_2_)
- Gametocytes of each type (*G*_1_,*G*_2_)
- Adaptive immunity to each type (*I*_1_,*I*_2_)
- Innate immunity (*Z*)

**Fig 12:**
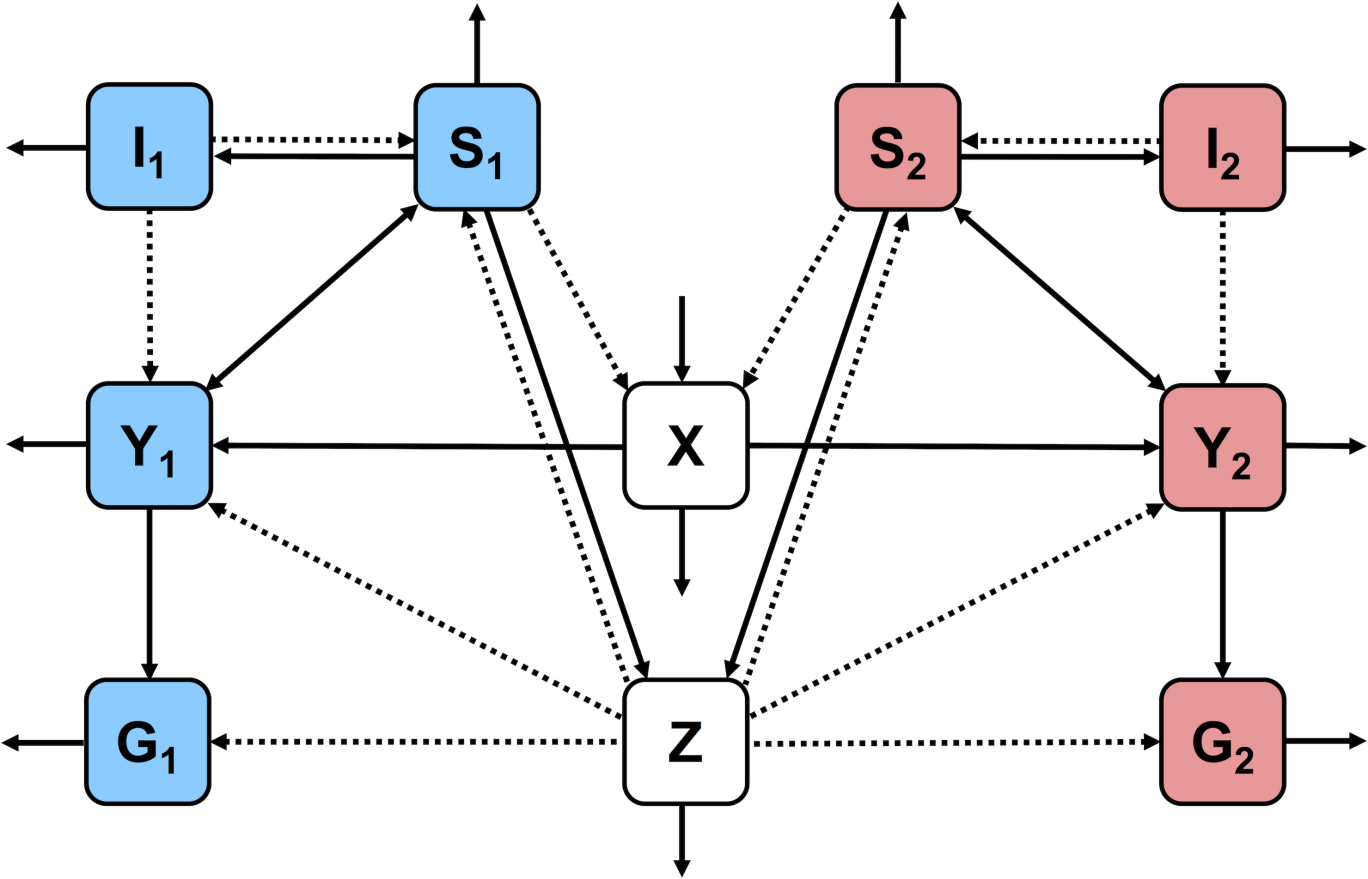
Compartment-style schematic of the within-host model. Strain-specific variables (infected RBCs, merozoites, gametocytes, and adaptive immunity) are color-coded, with variables relating to drug-sensitive and drug-resistant parasites shown in blue and red, respectively. Non-strain-specific variables (uninfected RBCs, innate immunity) are shown in white. Solid arrows indicate that one component increases or feeds into another, while dotted arrows indicate that one component reduces another. RBC, red blood cell.

Below are the ODEs for the within-host model. These equations, other than those governing acquired immunity, are similar to previous within-host models of malaria [48]. Subscripts *i* and *j* are used to indicate type-specific variables and parameters (if *i* = 1, then *j* = 2, and vice versa). The definitions of all parameters in Eqs 1–7 are given in Table 2 (parameter values in S1 Text), and Fig 12 shows a compartment-style schematic of the within-host model.

$$\frac{dX}{dt}=B-\alpha_{X}X-\beta X(S_{i}+S_{j})$$ (Eq 1)

$$\frac{dY_{i}}{dt}=\beta XS_{i}-\left(\frac{1}{1-e_{i}}\right)\;\alpha_{Y}Y_{i}-\gamma Y_{i}-\delta_{Z}ZY_{i}-\delta_{I}(I_{i}+\omega_{j}I_{j})Y_{i}$$ (Eq 2)

$$\frac{dS_{i}}{dt}=R\alpha_{Y}(1-\varphi_{i})Y_{i}-\alpha_{S}S_{i}-\beta XS_{i}-\delta_{Z}ZS_{i}-\delta_{I}(I_{i}+\omega_{j}I_{j})S_{i}$$ (Eq 3)

$$\frac{dG_{i}}{dt}=\gamma Y_{i}-\alpha_{G}G_{i}-\delta_{Z}ZG_{i}$$ (Eq 4)

$$\frac{dZ}{dt}=\zeta (1-Z)(S_{i}+S_{j})-\alpha_{Z}Z$$ (Eq 5)

$$\frac{dI_{i}}{dt}={\sigma I}_{i}\;\left(\frac{S_{i}+\lambda S_{j}}{\theta +S_{i}+{\lambda S}_{j}}\right)-\alpha_{I}J_{i}\;(1-\max(H_{i},{\lambda H}_{j}))\;I_{i}-\max(H_{i},H_{j})*\psi J_{i}I_{i}\;\left(1-(\frac{C_{i}^{k}}{C_{i}^{k}+A^{k}})\right)$$ (Eq 6)

$$\frac{dC_{i}}{dt}=H_{i}+\mu H_{j}$$ (Eq 7)

**Table 2:** Definitions of parameters in the within-host model (parameter values in S1 Text).

Parameter	Definition
*B*	Rate of production of new RBCs
*α*_*X*_	Death rate of uninfected RBCs
*β*	Rate of infection of RBCs by merozoites
*e*_*i*_	Increase in death rate due to antimalarial drug treatment, if applicable (*e*_*i*_ = *ε*_*i*_ if being treated; otherwise, *e*_*i*_ = 0)
*ε*_*i*_	Efficacy of antimalarial drug treatment against type *i*
*α*_*Y*_	Death rate of infected RBCs (hemolysis following parasite replication)
*γ*	Gametocyte formation rate
*δ*_*Z*_	Rate of killing by innate immunity
*δ*_*I*_	Rate of killing by adaptive immunity
*ω*_*j*_	Proportion of current type *i* antigens recognized by adaptive immunity to type *j*
*R*	Number of merozoites produced per infected RBC
*φ*_*i*_	Fitness cost (0 ≤ *φ*_*i*_ ≤ 1)
*α*_*S*_	Death rate of merozoites
*α*_*G*_	Death rate of gametocytes
*ζ*	Innate immunity activation rate
*α*_*Z*_	Innate immunity inactivation rate
*σ*	Maximum growth rate of adaptive immunity
*λ*	Overlap in fixed (nonvariant) antigens between strains
*θ*	Merozoite density at which growth rate of adaptive immunity is *σ*/2
*H*_*i*_	Infection status indicator for type *i* (*H*_*i*_ = 1 if infected with type *i*; otherwise, *H*_*i*_ = 0)
*ψ*	Maximum (initial) decay rate of adaptive immunity due to antigenic variation
*k*	Shape parameter for decay of adaptive immunity due to antigenic variation
*A*	Shape parameter for decay of adaptive immunity due to antigenic variation
*α*_*I*_	Decay rate of adaptive immunity in absence of infection
*J*_*i*_	*J*_*i*_ = 1 if *I*_*i*_ > baseline value; otherwise, *J*_*i*_ = 0
*C*_*i*_	Amount of exposure to variant antigens of current type *i*
*μ*	Overlap in variant antigens between strains

Abbreviation: RBC, red blood cell.

The dynamics described by the within-host model boil down to two essential processes: parasite replication and parasite–immune system interactions. Parasite replication is straightforward: merozoites invade RBCs, turning them into infected RBCs; infected RBCs either produce more merozoites or differentiate into gametocytes. RBCs are lost during this process but are continually replaced by new ones. Interactions between parasites and the immune system are more complicated. The model includes two types of immunity: innate and acquired (sometimes called adaptive). Innate immunity acts in a strain-transcending manner and is important for controlling parasite growth during the acute phase of the infection; acquired immunity is at least partly type specific (the degree of specificity is determined by the model parameters) and required for eventual clearance of the infection. Both innate and adaptive immune responses are triggered by parasites, and both decay in the absence of continued stimulation; however, the “on” and “off” rates for innate immunity are higher, resulting in a fast, self-limiting response. Acquired immunity takes longer to develop but decays very slowly and helps to limit parasite growth in subsequent infections. However, the dynamics of acquired immunity are complicated by antigenic variation, which is discussed in more detail below.

Antigenic variation is an evolved strategy for evasion of acquired immunity. The parasite has several dozen “variants” of an immunodominant surface protein but only expresses one variant at a time; when the immune system learns to recognize the current variant, the parasite switches to a different one [49,50]. Antigenic variation therefore interferes with recognition and killing by the adaptive immune system and helps to prolong the infection, which may not be cleared for several months (presumably when the variant repertoire has been exhausted). Thus, antigenic variation plays a key role in the dynamics of *P*. *falciparum* infections; however, explicitly modeling the process is extremely computationally demanding. Instead, our model implicitly incorporates antigenic variation by describing its effects on the dynamics of infection. Each switch to a novel variant interferes with recognition by the adaptive immune system, which makes the immune response less effective; this loss of effectiveness is mathematically indistinguishable from a loss of immune effectors. We therefore incorporate antigenic variation as a second decay term in the equations for adaptive immunity, which diminishes with the progress of the infection, since the variant repertoire is finite and eventually runs out.

### Between-host model

Unlike the within-host model, which is governed by deterministic equations, the between-host model is stochastic; as a result, multiple simulations with the same parameters and starting conditions will tend to yield similar but not identical results—unless the conditions favor highly divergent trajectories, such as scenarios that tip either toward extinction or toward epidemic spread.

The between-host model describes human-to-mosquito and mosquito-to-human transmission of parasites. Each human host is assigned to be bitten by a randomly chosen set of mosquitoes each day (the number of mosquitoes is based on the transmission intensity); these mosquitoes can infect and/or become infected by the human host when they feed. The probability of a mosquito becoming infected is determined by the total gametocyte density in the human host, using a gametocytemia-infectivity function originally described by Churcher and colleagues [51]. If the mosquito is determined to be infected, the number of gametocytes of each type picked up is determined by the individual gametocyte densities of drug-sensitive and drug-resistant parasites. If parasites are acquired, there is a latent period (10 days in the model) before the infection reaches the salivary glands and the mosquito becomes infective. Mosquitoes are allowed to acquire parasites from multiple hosts (though only one host per day); parasites acquired from different blood meals are tracked separately through the latent period and up to the point of transmission to a human host.

An infective mosquito has a constant probability of introducing parasites to the human host it feeds on. If this does occur, a fixed number of sporozoites is introduced; how many of these are drug-sensitive and drug-resistant depends on the ratio of the two types in the pool of gametocytes the mosquito originally acquired. Once sporozoites are transmitted to the human host, the infection goes through a latent period (i.e., the liver stage), which ends with merozoites being released into the bloodstream, at which point the parasites become subject to the within-host model.

### Parasite diversity

An aspect of the model with particularly broad ramifications is the diversity of the parasite population, particularly as it pertains to recognition by the adaptive immune system. In the simulations presented here, we assume a complete lack of population structure: all strains in the population, whether sensitive or resistant, are equally “related.” (However, we note that the design of the within-host model results in competition between sensitive and resistant strains being slightly weaker than competition between strains of the same type, particularly in hosts that have had only a few infections. This permits stable coexistence between the strains in settings in which transmission and cross-reactivity between different strains are both low—which is not unreasonable, since some degree of linkage between resistance mutations and antigenic loci is to be expected, particularly in low-transmission settings in which recombination rates are low) [52].

Relatedness of different strains is governed by the parameters *λ* and *μ*, which specify the fractions of fixed (nonvariant) and variant antigens shared by any two strains (for high cross-reactivity, we have *λ* = 0.7 and *μ* = 0.3; for low cross-reactivity, we use *λ* = 0.35 and *μ* = 0.15). These parameters determine cross-reactivity, or how much protection against one strain is conferred by previous exposure to a different strain [53]. This affects how quickly immunity is acquired: if cross-protection is minimal, then it may take many exposures to build up effective immunity [54, 55]. Cross-reactivity also determines the severity of immune-mediated competition between sensitive and resistant parasites, which affects the ability of resistant parasites to survive and spread in the face of competition from drug-sensitive parasites.

We note that in *P*. *falciparum*, genetic diversity is known to be greater in high-transmission settings [8], so strains in high-transmission areas might be expected to have lower cross-reactivity than strains in low-transmission regions. Thus, although it may be more straightforward to compare simulations that differ only in transmission intensity or only in cross-reactivity between strains, it may be important to compare high-transmission, low-cross-reactivity settings with low-transmission, high-cross-reactivity ones. Therefore, we present simulation outputs in sets of four—high and low transmission with high and low cross-reactivity—to make relevant comparisons while disentangling the effects of transmission intensity and cross-reactivity between strains.

### Antimalarial drug treatment

In the simulations presented here, antimalarial drug treatment is conditional only on the host being infected with parasites (above the extinction threshold, a total infected RBC density greater than 10^−4^). If a host is infected (and not already being treated), there is a fixed daily probability of beginning antimalarial treatment. If started, drug treatment is maintained for a fixed duration of 14 days, regardless of whether parasite clearance is achieved.

### Simulations

Simulated populations consisted of 400 human hosts and 12,000 mosquitoes; transmission intensity was determined by the rate of contact between humans and mosquitoes rather than the ratio of mosquitoes to humans. Simulations were run for a total duration of 8,000 days. Age was uniformly distributed with a maximum of 3,000 days; when hosts reached this limit, they were removed and replaced with naïve hosts of age zero. Drug-sensitive parasites were introduced at an initial prevalence of 10%, and the simulation was run for 3,000 days to allow the system to reach equilibrium before introducing drug-resistant parasites at a prevalence of 2%. Use of antimalarial drugs, when included, was initiated at the start of each simulation.

Certain parameters (human population size, starting prevalence of resistant parasites, host lifespan) were constrained by computational demands. A larger population size would considerably increase simulation run times and would not significantly change the results if the number of hosts initially infected with resistant parasites were held constant (this is because transmission is frequency dependent rather than density dependent). A lower starting prevalence of resistance, or stochastic introduction of resistance, would better simulate de novo emergence of resistance mutations; however, this would have significantly increased the number of simulations required, due to higher rates of extinction. Instead, we introduced resistant parasites into a fixed, slightly higher number of hosts in each simulation, representing hosts infected by a recently emerged drug-resistant mutant. Host lifespan was limited by the time required for the simulation to reach equilibrium before introducing resistant parasites. A longer lifespan would be expected to alter the distribution of immune states in a population, which would affect within-host dynamics (including competitive suppression and competitive release in mixed-strain infections) as well as between-host dynamics (including infection prevalence and the frequency of mixed infections). However, the qualitative differences between low- and high-transmission settings that underlie the results of our model would be expected to hold.

## Supporting information

S1 Text Details of the mathematical model, including parameter values. (PDF)

S1 Fig Introduction of resistant parasites into populations with varying levels of antimalarial drug use and fitness costs of resistance. Panels A–Q vary in treatment rate (daily probability of infected host starting treatment) and fitness cost (proportional reduction in within-host growth rate for the resistant type). Each four-part panel presents results for (i) low transmission/high cross-reactivity, (ii) high transmission/high cross-reactivity, (iii) low transmission/low cross-reactivity, and (iv) high transmission/low cross-reactivity. For all figures, solid lines and shaded areas show mean and range of 3 independent simulations, respectively. (Note that in some panels, such as C(ii), shading for one or more colors covers a wide area; this is generally caused by the resistant strain going extinct in some but not all of the replicate simulations.)
(TIF)

## Abbreviations

ACT: artemisinin-based combination therapy
EIR: entomological inoculation rate
MDA: mass drug administration
ODE: ordinary differential equation
RBC: red blood cell
