# Supplementary material for: Within-host competition can delay evolution of drug resistance in malaria
Source: PLoS Biol. 2018 Aug 21;16(8):e2005712. doi: 10.1371/journal.pbio.2005712 (PMC6103507; doi:10.1371/journal.pbio.2005712)
Supplement: S1 Text — (PDF) [file pbio.2005712.s001.pdf]

SUPPORTING INFORMATION

Within-host model

The within-host model describes the infection dynamics of two types of *Plasmodium falciparum* parasites – drug-sensitive (1) and drug-resistant (2). The model is comprised of a system of ordinary differential equations that describe the dynamics of the following components:

- Red blood cells (X)
- Infected red blood cells of each type (Y_1, Y_2)
- Merozoites (extracellular parasites) of each type (S_1, S_2)
- Gametocytes of each type (G_1, G_2)
- Adaptive immunity to each type (I_1, I_2)
- Innate immunity (Z)

In the following equations and explanations, we use subscripts i and j to denote type-specific variables and parameters; thus $(i, j) = (1,2)$ or $(2,1)$.
The differential equation for uninfected red blood cells is as follows:

$$\frac{dX}{dt} = \underbrace{B}_{\text{RBC production}} - \overbrace{\alpha_X X}^{\text{RBC death}} - \underbrace{\beta X(S_1 + S_2)}_{\text{Infection by merozoites}}$$

where B is the rate of production of new RBCs, α_X is the death rate of uninfected RBCs, and β is the rate of infection of RBCs by free merozoites (parameter values can be found in Table 1).

Infected RBCs of type i are described by the following equation:

$$\frac{dY_i}{dt} = \underbrace{\beta X S_i}_{\text{Infection of RBCs by merozoites}} - \underbrace{\overbrace{\left(\frac{1}{1 - e_i}\right)}^{\text{Death rate increase from antimalarial drug treatment}} \alpha_Y Y_i}_{\text{Infected RBC death}} - \overbrace{\gamma Y_i}^{\text{Gametocyte formation}} - \underbrace{\delta_Z Z Y_i}_{\text{Killing by innate immunity}} - \overbrace{\delta_I\big(I_i + \omega_j I_j\big)Y_i}^{\text{Killing by adaptive immunity}}$$

where α_Y is the background death rate of infected RBCs; in the absence of antimalarial drug treatment, $e_i = 0$ (making the death rate α_Y). If the host is being treated with antimalarial drugs, then $e_i = \varepsilon_i$ where ε_i represents the efficacy of drug treatment against type i. γ is the per capita rate of gametocyte formation. δ_I and δ_Z are the rates of killing by adaptive and innate immune responses, respectively. ω_j is the proportion of I_j (the adaptive immune response to type j) that is effective against type i. The relationship of ω_j to the antigenic overlap between different strains is discussed later on.

The equation for free merozoites of type i is:

$$\frac{dS_i}{dt} = \underbrace{R\alpha_Y \overbrace{(1-\varphi_i)}^{\text{Fitness cost}} Y_i}_{\text{Merozoite production by infected RBCs}} - \overbrace{\alpha_S S_i}^{\text{Merozoite death}} - \underbrace{\beta X S_i}_{\text{Merozoite invasion of RBCs}} - \overbrace{\delta_Z Z S_i}^{\text{Killing by innate immunity}} - \underbrace{\delta_I (I_i + \omega_j I_j) S_i}_{\text{Killing by adaptive immunity}}$$

where R is the burst size (number of merozoites released by a single infected red blood cell), φ_i is the fitness cost of type i (implemented as a reduction in burst size [1]), and α_S is the death rate of free merozoites. δ_I, δ_Z, and ω_j are as described above.

The equation for gametocytes of type i is below:

$$\frac{dG_i}{dt} = \underbrace{\gamma Y_i}_{\text{Gametocyte formation}} - \overbrace{\alpha_G G_i}^{\text{Gametocyte death}} - \underbrace{\delta_Z Z G_i}_{\text{Killing by innate immunity}}$$

where α_G is the death rate of mature gametocytes and γ and δ_Z are as described above. Due to the scarcity of gametocytes in the human host, we assume that adaptive immune responses to gametocytes are negligible. Therefore, "natural" death and killing by innate immunity are the only mechanisms by which gametocytes are eliminated in the model. Innate immunity is described by the following equation:

$$\frac{dZ}{dt} = \underbrace{\zeta(1-Z)(S_1+S_2)}_{\text{Innate immunity activation}} - \underbrace{\alpha_Z Z}_{\text{Innate immunity inactivation}}$$

where Z is considered the fraction of a fixed pool of innate immune effectors that are currently "activated." ζ is the activation rate of these effectors, and α_Z is the inactivation rate.

The dynamics of adaptive immunity to type i are described by the following equations:

$$\frac{dI_i}{dt} = \overbrace{\sigma I_i \left(\frac{S_i + \lambda S_j}{\theta + S_i + \lambda S_j}\right)}^{\text{Growth of adaptive immunity}} \overbrace{- \alpha_I J_i \left(1 - \max(H_i, \lambda H_j)\right) I_i}^{\text{Decay of adaptive immunity}}$$
$$\underbrace{-\max(H_1, H_2) * \psi J_i I_i \left(1 - \left(\frac{C_i^k}{C_i^k + A^k}\right)\right)}_{\text{"Loss" of adaptive immunity due to antigenic variation}}$$

$$\frac{dC_i}{dt} = \underbrace{H_i + \mu H_j}_{\text{Exposure to antigenic variants}}$$

The parameter σ is the maximum growth rate of the adaptive immune response and θ is the density of merozoites $(S_i + \lambda S_j)$ at which the growth rate is $\sigma/2$ (Figure 1). λ is the proportion of fixed (non-variant) antigens or epitopes that are shared between strains of types i and j. The contribution of type j merozoites to stimulation of I_i is proportional to this overlap.

Although both merozoites and infected RBCs will stimulate adaptive immune responses, the above equation is written such that only merozoites drive growth of adaptive immunity. This simplification is justified because infected RBCs and free merozoites maintain a relatively fixed ratio in the host, such that $S + Y \approx \rho S$; thus, this ratio ρ can simply be incorporated into the parameters σ and θ instead.

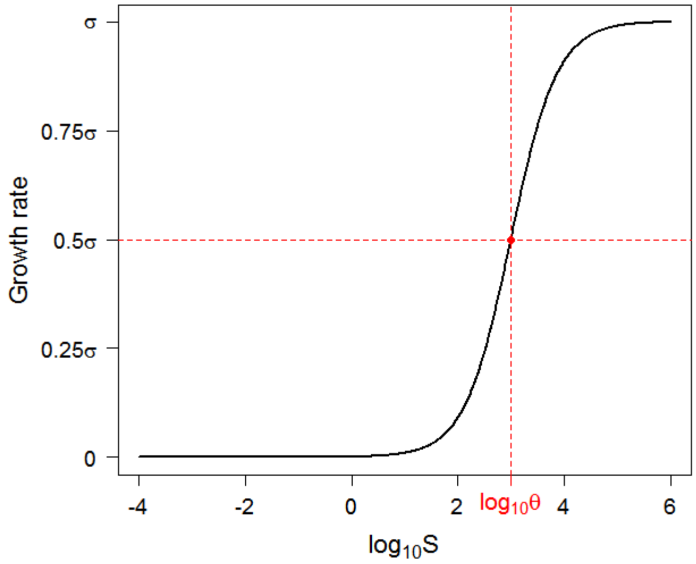

Figure 1. Growth rate of adaptive immunity $\left(\text{the positive term of } \frac{dI}{dt}\right)$ as a function of merozoite density (S). For simplicity, S here stands for $S_i + \lambda S_j$. Intersection of dashed lines identifies the point at which $S = \theta$ and the growth rate equals $\sigma/2$.

The equations for the adaptive immune responses each includes two decay terms, but the application of these terms depends on which type(s) are present in the host. The variable H_i is definied such that $H_i = 1$ if type i is present, and $H_i = 0$ otherwise. In addition, the variable J_i ensures that I_i does not decline below the baseline value I_N: $J_i = 0$ if $I_i \leq I_N$, and $J_i = 1$ otherwise.

The first decay term, with coefficient $\left(1 - \max\left(H_i, \lambda H_j\right)\right)$, is simply the slow, exponential decline of adaptive immunity in the absence of continued stimulation (the half-life being measured in years). If type i is present, this term simplifies to zero. If both types are absent, the term simplifies to $-\alpha_I I_i$. If type i is absent but type j is present, and as long

as $I_{ij} > I_N$, the applicable decay term is $-\alpha_I(1-\lambda)$. The parameter λ is the proportion of antigens that are shared betweens strains of type i and type j; therefore, only the non-overlapping proportion $(1-\lambda)$ decays when type i is absent but type j is present.

The second decay term, with coefficient $\max(H_1, H_2)$, is applied whenever the host is infected with either type. When the host is infected (with either type, or both types), and as long as $I_i > I_N$, the applicable decay term is $-\psi I_{ij}\left(1-\left(\frac{C_{ij}^k}{C_{ij}^k+A^k}\right)\right)$. As described in more detail below, C_i increases with time, the fraction $\left(\frac{C_{ij}^k}{C_{ij}^k+A^k}\right)$ approaches 1, and the decay rate approaches zero. The relationship between C_i and the decay rate is depicted in Figure 2.

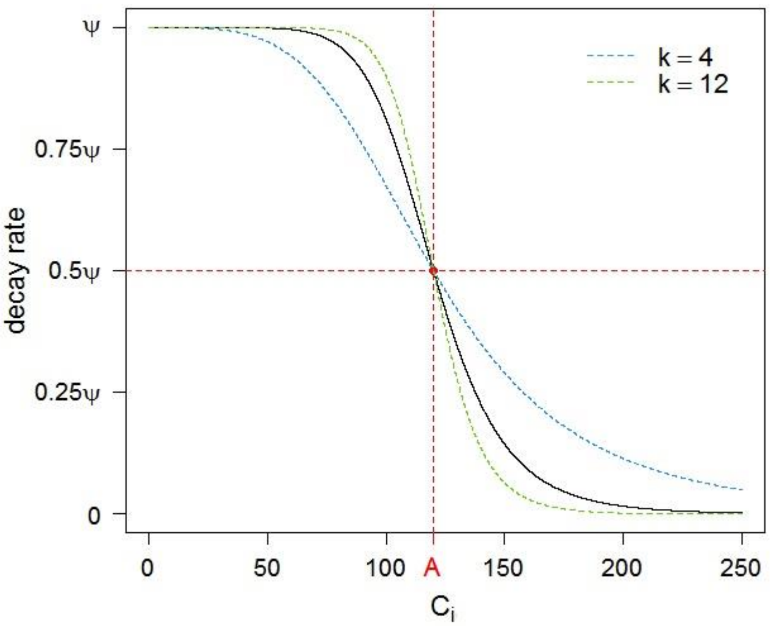

Figure 2. Decay rate of I_i as a function of C_i (black line). Intersection of dashed red lines indicates the point where $C = A$ and the decay rate equals $\psi/2$. Dashed blue and green lines show what the function looks like for alternative values of k ($k = 4$, blue; $k = 12$, green; black line with $k = 8$).

The function of this second decay term is to approximate the process of immune evasion through antigenic variant switching. Variant switching is thought to be stochastic in nature, although the degree of randomness is not known. For what follows, we assume that switching is at least approximately random (not heavily biased toward particular switching patterns), and that the sequential appearance of individual variants is driven by selection from adaptive immunity [2].

P. falciparum has a large, but finite, pool of variant antigens to switch through; for example, the size of the *var* gene repertoire is generally around 60 variants. If variant switching is approximately random, the time it takes to “find” a variant that is not recognized by the adaptive immune response is primarily a function of how many variants are already recognized. Early in the infection, almost any variant will not be recognized, so “escape” through switching should happen rapidly. However, when most variants have

been seen by the immune system, it will take many more random switches to find one that has not been seen before. Assuming random switching, the number of switches to find a novel variant follows a geometric distribution, with mean $\frac{1-p}{p}$ where p is the proportion of variants that have not been seen yet (Figure 3).

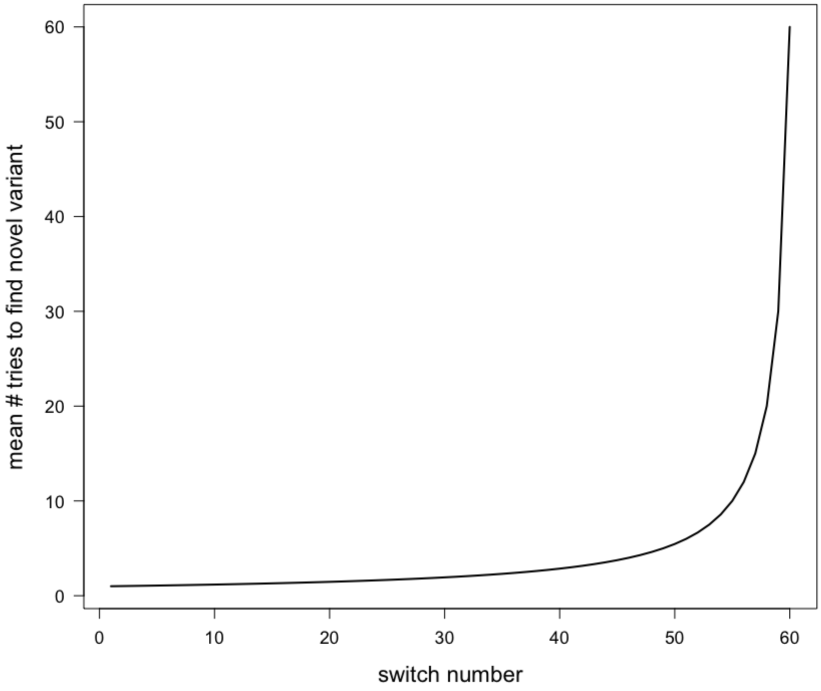

Figure 3. The mean number of antigenic variants that must be 'tried' before finding a variant the immune system has not seen, as a function of the number of variants that have already been seen (out of 60 total variants).

Rather than explicitly model the dynamics of variants and variant-specific immune responses, we use this hypothesized relationship between the number of variants already seen and the time required to "find" a novel variant to implicitly model the process of antigenic variation. The switch to a novel variant impairs the ability of the adaptive immune system to recognize and kill parasites; this loss of effectiveness is mathematically indistinguishable from a loss of immune effectors, and can thus be represented by a decay term in the equation for adaptive immunity.

As described above, novel variants should be found rapidly at the start of an infection, but much more slowly as the pool of variants is exhausted. Therefore, the rate of decay of adaptive immunity should be high initially and decrease as the infection progresses. The variable C_i exists to track the "progress" of an infection – i.e. how much of the variant repertoire has been "seen" by the adaptive immune system. We assume that only one variant is expressed at any given time, and therefore C_i increases linearly with time. However, different strains can have variants in common, and any shared variant expressed by one has been "used up" for all. Therefore, type j contributes to the increase of C_i over time at a rate that is proportional to the overlap in the variant repertoires of strains of types i and j (the parameter μ).

Parasite diversity and acquired immunity

For the purposes of this model, we assume that the parasite population is comprised of a virtually infinite pool of strains, such that every exposure is considered to be a new strain. Strains are classified phenotypically into drug-sensitive and drug-resistant 'types' but there is assumed to be no underlying population structure. Any two strains (whether of the same type or different types) are assumed to have a fixed amount of overlap in the proteins/antigens that are visible to the adaptive immune system; the amount of overlap determines the extent of cross-reactivity been strains. At the population level, greater cross-reactivity reduces the number of exposures required to reach a given 'degree' of acquired immunity. At the within-host level, greater cross-reactivity can increase the severity of immune-mediated 'apparent competition' in which the immune response generated by one strain nevertheless has a negative effect on both strains.

The overlap between strains is governed by two parameters. λ is the proportion of fixed (non-variant) antigens that are shared between any two strains, while μ is the proportion of variant antigens (such as *PfEMP1)* that are shared. There are two reasons that overlap of fixed antigens and overlap of variant antigens are considered separately. The first is simply that overlap in variant repertoires can be quite low (sometimes approaching zero). The second is that fixed and variant antigens have different effects on the dynamics of immunity. When fixed antigens are shared, it has the effect of boosting the immune response, whereas when variant antigens are shared, it hastens the exhaustion of each strain's variant repertoire. The logic is as follows: suppose two strains in the same host share a particular antigenic variant. When one of the strains expresses this variant, the adaptive immune system mounts a response against it. However, when the other strain switches to expressing this variant, the specific immunity acquired from previous exposure will not contribute much to control of parasite growth; instead, it will simply exert selection for other variants that are not yet recognized. Thus, any variant expressed by either strain has been 'used up' for both, which decreases the time until both strains run out of novel variants.

As mentioned above, we assume that any two strains share an equal proportion (λ) of their non-variant antigens; the proportion shared by n strains is λ^{n-1}. When a new strain infects a host that has previously encountered n strains, the host's immune system will recognize a proportion $(1-(1-\lambda)^n)$ of the new strain's antigens. Thus, in the model, every time a new strain of type i is introduced to a host, the adaptive immune response I_i is multiplied by the proportion of the new strain's antigens that are recognizable based on past exposures: $I_i \times (1-(1-\lambda)^{n_i})$ where n_i is the number of past encounters with type i.

Something similar is done for the variable C_i, which tracks how much of the current strain's antigenic variant repertoire the immune system has seen. When a new strain of type i is introduced, C_i is multiplied by the proportion of the new strain's variants that have been seen before: $C_i \times (1-(1-\mu)^{n_i+n_j})$ where n_i and n_j are the number of past exposures to type i and type j, respectively.

Finally, the number of previous exposures affects the degree to which each type is affected by the acquired immune response to the other type. The rate of killing of type i by

acquired immunity to type j is proportional to ω_j where $\omega_j = 1 - (1 - \lambda)^{n_j}$ (n_j is as defined above).

Human-mosquito contact and parasite transmission

Every day, each human host is assigned to be bitten by a number of mosquitoes that is drawn from a Poisson distribution with mean b. Each mosquito bites only one host per day, and which mosquitoes bite on any given day is random (mosquitoes can bite on sequential days but do not necessarily do so).

The probability that a mosquito is infected upon feeding on a host is determined by a function described by Churcher et al. [3]:

$$P = \left(1 - \left(1 + \frac{(G_1 + G_2)}{2d}\right)^{d-1}\right)\left(g_0 + g_1 \exp\left(-g_2 \exp\left(-g_3(G_1 + G_2)\right)\right)\right)(1 + p + q)$$

where $p = \begin{cases} 0 & \text{if } (Y_1 + Y_2) < 100 \\ f_1 & \text{if } 100 \leq (Y_1 + Y_2) < 1000 \\ f_2 & \text{if } (Y_1 + Y_2) \geq 1000 \end{cases}$ and $q = \begin{cases} 0 & \text{if age} < 5 \text{ years} \\ 1 & \text{otherwise} \end{cases}$

If a mosquito is determined to be infected, the number of gametocytes of type i picked up is drawn from a Poisson distribution with mean $G_i * V$ where $G_i =$ type i gametocytes/μL and V is the volume of a mosquito blood meal in μL. (Draws of zero gametocytes are disallowed because the number of mosquitoes infected is pre-determined by the gametocyte density-infectivity function shown above.)

Infection in the mosquito has a latent period of y days. After the latent period ends (simulating the appearance of sporozoites in the salivary glands), the mosquito becomes infective. When an infective mosquito bites a host, there is a fixed probability f that sporozoites are transmitted to the host; if this occurs, the mosquito introduces n sporozoites to the host. If m_i gametocytes of type i were originally, then drawing from a binomial distribution with size n and probability $m_1/(m_1 + m_2)$ determines the number of sporozoites of each type transmitted. If parasites from K blood meals have reached the infective stage, assuming m_{ix} is the number of type i gametocytes acquired from blood meal x ($1 \leq x \leq K$), then a draw of size n is made from a multinomial distribution with probability $\left(\frac{1}{K}\right)\left(\frac{m_{ix}}{m_{ix}+m_{jx}}\right)$ for type i from blood meal x. The sporozoites from different blood meals are considered separately for the purposes of tracking the human host's exposure to strains of each type. If the host receives sporozoites of type i that were derived from 3 different blood meals, then the 'strain exposure count' for type i increases by 3, even though the parasites were introduced by the same mosquito. However, when a mosquito acquires gametocytes from a host, the gametocytes of each type are considered to constitute one strain, even if they were derived from multiple introductions.

Infection in the human host also has a latent period of w days, which simulates the liver stage of the infection. At the end of the latent period, M merozoites are released for each sporozoite introduced w days before and are added to the circulating merozoites

tracked by the within-host model. At this point, the host's 'exposure count' for each type is updated to reflect the number of strains represented among the newly-emerged parasites.

Populations and turnover

The human population consists of N_H hosts with ages uniformly distributed between zero and the human lifespan, a. A host that reaches age a is replaced by a host of age zero with a 'clean slate' – no current infection or latent infection, no history of infection, and no immunity. The mosquito population is similar (except for having a much higher rate of turnover); the population consists of N_M mosquitoes that are evenly divided between ages zero to z (the mosquito lifespan), and each day the oldest mosquitoes are removed and replaced with new mosquitoes of age zero.

Treatment

The simulated use of antimalarial drugs is flexible in a few ways. Treatment can be made conditional on the total parasite density $(Y_1 + Y_2)$ exceeding a threshold, which can simulate treating only symptomatic infections or only infections detectable by standard diagnostic methods; alternatively, treatment can be administered to any infected host (by setting the threshold parasite density to zero) or to any host regardless of infection status (by setting the threshold to -1). Antimalarial drug use can be started in the middle of a simulation, to simulate introduction of a drug into a population at equilibrium. Treatment can also be restricted to start only on certain days, which can simulate mass drug administration (MDA) or mass screening and treatment (MSAT) where antimalarial drugs are administered en masse at regular intervals. Not all of these options are used in the simulations presented, but they provide opportunities to further explore the fate of drug resistance in scenarios not considered here.

Table 1. Default model parameters; those varied for the simulations presented in this work are noted as such.

Variable or Parameter	Value	Definition
B	$(5/12) \times 10^5$	Production rate of new RBCs (per µL per day)
α_X	$1/120$	Death rate of uninfected RBCs
β	2.4×10^{-6}	Infection rate (merozoite invasion of RBCs)
ε_i	$\varepsilon_1 = 0.95,\ \varepsilon_2 = 0$	Antimalarial drug efficacy against type i
α_Y	0.5	Death rate of infected RBCs (hemolysis following parasite replication)
γ	0.02	Gametocyte formation rate
δ_I	4	Rate of killing by adaptive immunity
ω_i	depends on infection history	Effect of adaptive immunity to type i on other type
δ_Z	4	Rate of killing by innate immunity
R	16	Burst size (merozoites per infected erythrocyte)
φ_i	varies	Fitness cost (growth reduction) of type i
α_S	48	Death rate of free merozoites
α_G	0.0625	Death rate of gametocytes

ζ	3×10^{-5}	Growth rate of innate immunity
α_Z	0.5	Decay rate of innate immunity
σ	1	Maximum growth rate of adaptive immunity
θ	10^3	Shape parameter (adaptive immunity growth curve)
ψ	0.1	Decay rate of adaptive immunity due to antigenic escape
A	120	Shape parameter (adaptive immunity decay due to antigenic escape)
k	8	Shape parameter (adaptive immunity decay due to antigenic escape)
α_I	10^{-3}	Background decay rate of adaptive immunity
I_N	10^{-3}	Starting value of adaptive immunity to each type
Ω	10^{-4}	Extinction threshold (infected RBC density)
L	14	Duration of treatment (days)
z	14	Mosquito lifespan (days)
y	10	Latent period in mosquito (days)
w	12	Latent period (liver stage) in humans
V	2	Blood meal volume (µL)
n	12	Number of sporozoites introduced by each mosquito bite
M	10^4	Number of merozoites produced per sporozoite (liver stage)
a	3000	Human lifespan (days)
N_H	400	Human population size
N_M	1.2×10^4	Mosquito population size
b	Varies	Mean number of mosquito bites per person per day
λ	0.7 or 0.35	Proportion of fixed antigen epitopes shared between strains
μ	0.3 or 0.15	Proportion of variant antigen epitopes shared between strains
Y_{TR}	>0	Minimum infected RBC density required for detection and treatment
p_{TR}	varies	Probability host treated if other conditions met
τ	1	Interval (days) at which hosts are screened and treatment initiated
ι_i	$\iota_1 = 0.1, \iota_2 = 0.02$	Initial fraction of hosts infected with type i
d	0.0446	Mosquito infection function parameter [3]
f_1	0.181	Mosquito infection function parameter [3]
f_2	0.881	Mosquito infection function parameter [3]
f_3	0.0904	Mosquito infection function parameter [3]
g_0	0.0382	Mosquito infection function parameter [3]
g_1	0.165	Mosquito infection function parameter [3]
g_2	51.4	Mosquito infection function parameter [3]
g_3	0.0129	Mosquito infection function parameter [3]

References

1. Mideo N, Savill NJ, Chadwick W, Schneider P, Read AF, Day T, et al. Causes of variation in malaria infection dynamics: insights from theory and data. Am Nat. 2011;178(6):E174-88.
2. Recker M, Nee S, Bull PC, Kinyanjui S, Marsh K, Newbold C, et al. Transient cross-reactive immune responses can orchestrate antigenic variation in malaria. Nature. 2004;429(6991):555-8.
3. Churcher TS, Bousema T, Walker M, Drakeley C, Schneider P, Ouedraogo AL, et al. Predicting mosquito infection from Plasmodium falciparum gametocyte density and estimating the reservoir of infection. Elife. 2013;2:e00626.
