# Supplementary figures and images for: Within-host competition can delay evolution of drug resistance in malaria
Source: PLoS Biol. 2018 Aug 21;16(8):e2005712. doi: 10.1371/journal.pbio.2005712 (PMC6103507; doi:10.1371/journal.pbio.2005712)

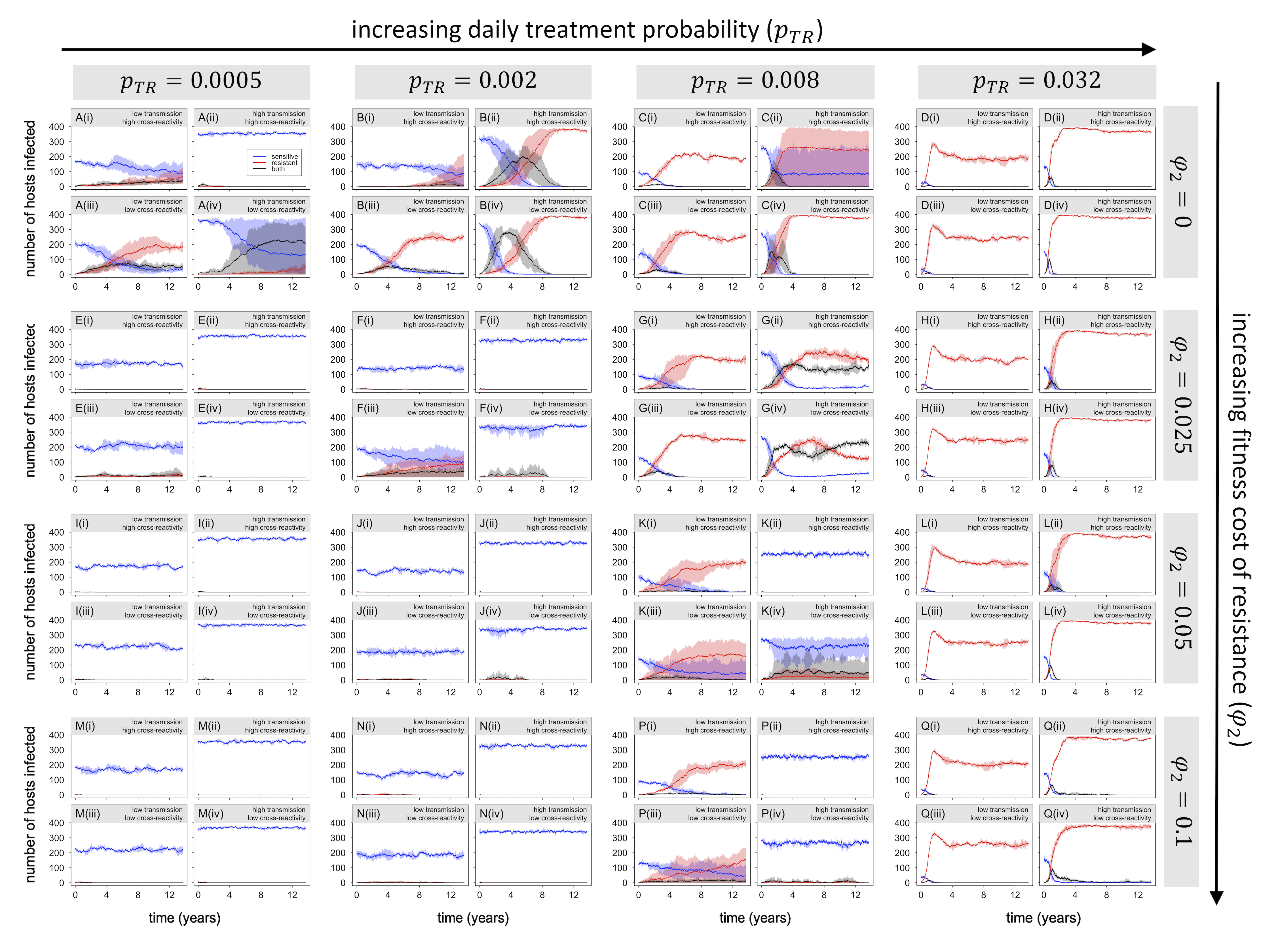

Supplement: S1 Fig — Panels A–Q vary in treatment rate (daily probability of infected host starting treatment) and fitness cost (proportional reduction in within-host growth rate for the resistant type). Each four-part panel presents results for (i) low transmission/high cross-reactivity, (ii) high transmission/high cross-reactivity, (iii) low transmission/low cross-reactivity, and (iv) high transmission/low cross-reactivity. For all figures, solid lines and shaded areas show mean and range of 3 independent simulations, respectively. (Note that in some panels, such as C(ii), shading for one or more colors covers a wide area; this is generally caused by the resistant strain going extinct in some but not all of the replicate simulations.) (TIF) [file pbio.2005712.s002.tif]
